# T-type, but not L-type, voltage-gated calcium channels are dispensable for lymphatic pacemaking and spontaneous contractions

**DOI:** 10.1038/s41598-019-56953-3

**Published:** 2020-01-09

**Authors:** Kim H. T. To, Peichun Gui, Min Li, Scott D. Zawieja, Jorge A. Castorena-Gonzalez, Michael J. Davis

**Affiliations:** Department of Medical Pharmacology and Physiology, University of Missouri School of Medicine, Columbia, Missouri 65212 USA

**Keywords:** Cardiovascular biology, Circulation

## Abstract

The spontaneous contractions of collecting lymphatic vessels provide an essential propulsive force to return lymph centrally. These contractions are driven by an intrinsic electrical pacemaker, working through an unknown underlying ionic mechanism that becomes compromised in some forms of lymphedema. In previous studies, T-type voltage-gated Ca^2+^ channels (VGCCs) were implicated in this pacemaking mechanism, based on the effects of the reputedly selective T-type VGCC inhibitors mibefradil and Ni^2+^. Our goal was to test this idea in a more definitive way using genetic knock out mice. First, we demonstrated through both PCR and immunostaining that mouse lymphatic muscle cells expressed Ca_v_3.1 and Ca_v_3.2 and produced functional T-type VGCC currents when patch clamped. We then employed genetic deletion strategies to selectively test the roles of each T-type VGCC isoform in the regulation of lymphatic pacemaking. Surprisingly, global deletion of either, or both, isoform(s) was without significant effect on either the frequency, amplitude, or fractional pump flow of lymphatic collectors from two different regions of the mouse, studied *ex vivo*. Further, both WT and Ca_v_3.1^−/−^; 3.2^−/−^ double knock-out lymphatic vessels responded similarly to mibefradil and Ni^2+^, which substantially reduced contraction amplitudes and slightly increased frequencies at almost all pressures in both strains: a pattern consistent with inhibition of L-type rather than T-type VGCCs. Neither T-type VGCC isoform was required for ACh-induced inhibition of contraction, a mechanism by which those channels in smooth muscle are thought to be targets of endothelium-derived nitric oxide. Sharp intracellular electrode measurements in lymphatic smooth muscle revealed only subtle, but not significant, differences in the resting membrane potential and action potential characteristics between vessels from wild-type and Ca_v_3.1^−/−^; 3.2^−/−^ double knock-out mice. In contrast, smooth-muscle specific deletion of the L-type VGCC, Ca_v_1.2, completely abolished all lymphatic spontaneous contractions. Collectively our results suggest that, although T-type VGCCs are expressed in mouse lymphatic smooth muscle, they do not play a significant role in modulating the frequency of the ionic pacemaker or the amplitude of spontaneous contractions. We conclude that the effects of mibefradil and Ni^2+^ in other lymphatic preparations are largely or completely explained by off-target effects on L-type VGCCs, which are essential for controlling both the frequency and strength of spontaneous contractions.

## Introduction

The spontaneous contractions of collecting lymphatic vessels propel lymph centrally to account for 2/3 of peripheral lymph flow^1,2^. These rapid, large-amplitude contractions are analogous to twitch contractions of cardiac and skeletal muscle and are particularly important for moving lymph uphill against the adverse hydrostatic gradients that exist in dependent extremities. Lymphatic contractions are triggered by action potentials (APs) in lymphatic smooth muscle cells (LMCs), with a single AP typically evoking a single twitch contraction. Although the underlying ionic mechanisms of the AP in LMCs are still being debated, it is well established that inward current during the AP is carried by voltage-gated calcium channels (VGCCs), with a component in some species contributed by voltage-gated sodium channels^3–7^. Both spontaneous APs and their associated contractions are blocked by dihydropyridines (DHPs) in rat, pig, cow, guinea pig, sheep and human lymphatic vessels^8–10^, suggesting a primary role for L-type VGCCs (Ca_v_1.2), which contain a highly-conserved DHP binding site^11^. The dependence of spontaneous lymphatic contractions on Ca_v_1.2 channels is presumably explained by that channel accounting for the majority of the calcium entry that controls the activation of regulatory myosin light chains, similar to its role in myogenic- and agonist-induced constriction of arterial smooth muscle^12–15^.

Recently, two studies have implicated T-type VGCCs as contributors to pacemaking in lymphatic vessels. T-type VGCCs have a more negative activation threshold and inactivate more rapidly than L-type VGCCs, making them potential candidates for being triggered first by depolarizing pacemaker potentials^16^, followed by activation of L-type VGCCs^17^. Of the three known T-type VGCCs, two isoforms, Ca_v_3.1 (*CANCA1G*, α1 G) and Ca_v_3.2 (*CANCA1H*, α1 H), are expressed in a number of different tissues, including cardiac muscle^18,19^ and vascular smooth muscle^20–23^; in contrast, the third isoform, Ca_v_3.3 (*CANCA1I*, α1I), is expressed primarily in neurons^24,25^. Electrophysiological evidence supports an important role for Ca_v_3.3 in neuronal pacemaking^26–29^ and for Ca_v_3.1 in cardiac pacemaking^30,31^. Although Ca_v_3 channels may not be involved in the “pacemaking” per se of arteries/arterioles, which are typically characterized by chronic tone generation, stable resting membrane potentials and slow, pressure-dependent contractions rather than twitch contractions, other important roles for Ca_v_3 channels in arterial smooth muscle have been uncovered recently^20,21,23,32,33^. For these reasons it is reasonable to expect that Ca_v_3 channels could be important contributors to voltage-gated Ca^2+^ influx in lymphatic smooth muscle. In support of this idea, Beckett *et al*.^8^ found that Ni^2+^, at a concentration (100 μM) often used to inhibit Ca_v_3.1 in other tissues^21,34^, led to a selective reduction in the frequency of AP firing in LMCs from sheep mesentery, whereas the L-type VGCC inhibitor nifedipine (1 μM, a concentration higher than typically needed to selectively block Ca_v_1.2) led to a reduction in AP amplitude without a concomitant change in frequency; however, over time both compounds eventually produced a complete block of spontaneous AP activity, i.e. inhibiting both AMP and FREQ, which is suggestive of an off-target effect on L-type VGCCs. A subsequent study of rat lymphatic vessels by Lee *et al*.^35^, using both pressure- and wire-myography, found that Ni^2+^ (100 μM) and another reputed T-type VGCC inhibitor, mibefradil (0.1 μM), selectively reduced the AP firing rate, whereas nifedipine (0.3 μM) reduced contraction amplitude with essentially no effect on frequency. The collective conclusion of those two studies was that Ca_v_3 channels determine the rate of AP firing (i.e. the contraction rate) while Ca_v_1.2 channels determine contraction amplitude^8,35^.

The interpretation of the evidence that T-type VGCCs selectively regulate the frequency of lymphatic pacemaking depends critically on the specificity of the inhibitors Ni^2+^ and mibefradil. Unfortunately, both compounds are known to have off-target effects on Ca_v_1.2^36^ as well as on other ion channels^37–39^. Based on studies of recombinant Ca_v_3 channels in heterologous expression systems, the IC_50_ for Ni^2+^ is 6–12 μM for Ca_v_3.2, 167–250 μM for Ca_v_3.1, and 324 μM for Ca_v_1.2^40,41^, suggesting that Ni^2+^ may be useful only for selective blockade of Ca_v_3.2 channels^23^. Indeed, higher concentrations of Ni^2+^ are known to inhibit both Ca_v_3 and Ca_v_1.2 channels in native cells such as ventricular myocytes^42^. Mibefradil may be even less selective than Ni^2+^ because the IC_50_ for mibefradil for recombinant T-type VGCCs (0.87 μM) is quite similar to that for recombinant Ca_v_1.2 channels (1.4 μM)^43^. Indeed, a growing number of studies have questioned the specificity of mibefradil for T- over L-type VGCCs in native tissues^34,36^. The observation that the antihypertensive effect of systemic mibefradil administration is eliminated in animals with smooth-muscle specific deletion of Ca_v_1.2^36^ argues strongly that its effect on arterial tone is mediated completely by its action on L-type VGCCs. In addition, both Ni^2+^ and mibefradil inhibit other ion channels, such as KATP and delayed rectifier K^+^ channels, which also may regulate LMC membrane potential and thereby influence the activation or inactivation states of other channels involved in generating pacemaker potentials^36–39,44,45^.

The clinical relevance of determining the ionic currents underlying the lymphatic AP relates to the impairment of the pacemaking mechanism in patients with lymphedema. Olszewski measured hydrostatic pressures in lymphatic vessels in the legs of patients with chronic lymphedema and noted that “overloading of lymphatics with an excess of continuously produced lymph brings about dilatation of vessels” and “stretched lymphatics [that, compared to lymphatics in healthy patients,] do not generate effective pressures sufficient to propel lymph”^46–48^. These observations point to problems with impaired lymphatic smooth muscle contraction strength and/or pacemaking mechanism that potentially might be corrected pharmacologically. Eventual therapeutic targeting of ionic dysfunction in LMCs will require knowledge of the specific VGCC isoforms expressed in human LMCs as well as the development of selective modulators of those channels.

To address the roles of VGCCs in LMCs in a way that does not rely on poorly selective inhibitors, we used genetic approaches to delete specific L- or T-type VGCC isoforms from mouse LMCs. Using popliteal afferent lymphatics and inguinal-axillary lymphatics (IALs) as models of robustly contracting peripheral lymphatic vessels, we first identified which Ca_v_3 and Ca_v_1.2 isoforms were expressed, used patch clamp methods to verify the presence of functional Ca_v_3 channels based on their kinetics and voltage dependency, and then studied the contractile function and characteristics of the LMC action potential in lymphatic vessels from mice with global- or smooth muscle-specific deletions of those isoforms. These properties (including pacemaking frequency and contraction amplitude) were studied using *ex vivo*, pressurized lymphatic vessels from WT mice and mice deficient in Ca_v_3.1, Ca_v_3.2 or both Ca_v_3.1/Ca_v_3.2. We also tested lymphatic vessels from mice with smooth-muscle-specific deletion of Cav1.2. Our results lead to the surprising conclusion that T-type VGCCs do *not* play any significant role in controlling the pacemaking frequency or contributing to the amplitude of lymphatic spontaneous contractions, while L-type VGCCs are essential for both.

## Materials and Methods

### Animals

Male Sprague-Dawley rats were purchased from Harlan Laboratories (Indianapolis, IN). C57BL/6 J wild-type (WT) were purchased from Jackson Laboratory (Bar Harbor, ME, USA). Ca_v_3.1^−/−^ mice^49^ on the C57BL/6J background were a gift from Hee-Sup Shin (Korea Institute of Science and Technology) and Jeffrey Molkentin (University of Cincinnati), and the mice were rederived at MMRRC (Columbia, MO). Ca_v_3.1^−/−^ mice were originally generated by deleting most of the exon encoding amino acid residues 82–118 that comprise the N-terminus of *CANCA1G*^49^. The initial set of 24 Ca_v_3.1^+/−^ offspring from three C57BL/6J recipient mice were interbred to obtain Ca_v_3.1^−/−^ mice, which were genotyped to verify disruption of the Ca_v_3.1 gene; the lack of Ca_v_3.1 message in brain homogenate was further confirmed by RT-PCR. Ca_v_3.2^−/−^ mice were originally generated by deletion of exon 6 of *CANCA1H*^50^; we obtained them from JAX (B6; 129-Cacna1h,tm1Kcam./J; #013770), bred on the C57BL/6J background. A set of primers described by Lin *et al*.^51^ directed to the exon 6 region of *CANCA1H* (see Table 1) detected the absence of full length Ca_v_3.2 in brain homogenate. We also confirmed a known phenotype of 12-week old Ca_v_3.2^−/−^ mice described by Lin *et al*.^51^: a narrowing of the trachea due to malformed cartilage that results in audible “chirping” sounds during normal respiration. Ca_v_3.1^−/−^ mice were bred with Ca_v_3.2^−/−^ mice to generate Ca_v_3.1^−/−^; Ca_v_3.2^−/−^ double KO mice after three generations. SMMHC-CreER^T2^ mice (B6.FVB-Tg(Myh11-cre/ERT2)1Soff/J) were obtained from Stefan Offermanns (Max-Planck Institute, Bad Neuheim) and bred with Ca_v_1.2^fl/fl^ mice (*Cacna1c*^tm3Hfm^/J; #024714), purchased from JAX, to generate SMMHC-CreER^T2^; Ca_v_1.2^fl/fl^ mice. The offspring were injected with tamoxifen (i.p., 1 g/ml) for 5 days and allowed to recover for 10–12 days before testing, as previously described^14^. SMMHC-Cre^GFP^ mice (strain # 07742) on the C57BL/6J background were obtained from JAX; Prox1^GFP^ mice were a gift of Young Hong, University of Southern California; these two strains were used for some patch clamp and PCR protocols. All genotypes were verified by PCR. Mice (18–25 g) were studied at 5–10 weeks of age from either sex upon availability, except in the case of SMMHC-CreER^T2^ mice in which only male mice were used as the transgene is located on the Y-chromosome. All animal protocols were approved by the University of Missouri Animal Care and Use Committee and conformed to the National Institutes of Health’s *Guide for the Care and Use of Laboratory Animals (8*^*th*^ *edition, 2011)*.

**Table 1:** Primers used in end-point PCR.

Target	Accession number	Primer sequence (5′ to 3′)	Size
Ca_v_3.1 external	NM_009783	F: 5′-TGT GGA AAT GGT GGT GAA GA-3′	150 bp
		R: 5′-ACT GCG GAG AAG CTG ACA TT -3′	
Ca_v_3.1 internal		F: 5′-GAT GGT CGC TTT GGG TAT CT-3′	131 bp
		R: 5′-ACT GCG GAG AAG CTG ACA TT-3′	
Ca_v_3.2 external	NM_021415	F: 5′- TTC ATT GTC ATG GCT GGC AT-3′	289 bp
		R: 5′- TCA GGT TGT TGT TCC TGA CG-3′	
Ca_v_3.2 internal	NM_021415	F: 5′-TAC TCT CTG GAC GGA CAC AA-3′	262 bp
		R: 5′-TCA GGT TGT TGT TCC TGA CG-3′	
Ca_v_3.3 external	NM_001044308	F: 5′-GAT CCT GCA GGT CTT TGA TGA-3′	239 bp
		R: 5′-GAA CAC GGT TGA TGG CTT TG-3′	
Ca_v_3.3 internal		F: 5′-TGG GCA TTT TTG GCA AGA A-3′	129 bp
		R: 5′-CAG TGC GGA TGG CTG ACA-3′	
Ca_v_1.2	NM_001159533	F: 5′- CAG GAG GTG ATG GAG AAG CCA -3′	315 bp
		R: 5′-CTG CAG GCG GAA CCT GTT GTT-3′	
Ca_v_1.3 external	NM_028981	F: 5′- TGC TGT GAG GAC GAC AGC TCT CCC A -3′	342 bp
		R: 5′- TAG GCC TGC AAC GGC CAT GAT CTG C -3′	
Ca_v_1.3 internal		F: 5′- CAG CTC CAT GGA CTT TGA GAG -3′	274 bp
		R: 5′- TAG GCC TGC AAC GGC CAT GAT CTG C -3′	
Smooth muscle α-actin	NM_007392	F: 5′-GTG AAG AGG AAG ACA GCA CAG-3′	146 bp
		R: 5′-GCC CAT TCC AAC CAT TAC TCC-3′	
eNOS	NM_008713	F: 5′-CTG CCA CCT GAT CCT AAC TTG-3′	143 bp
		R: 5′-CAG CCA AAC ACC AAA GTC ATG-3′	
**Exons of Ca**_**v**_**1.2**			
exon1A	NM_009781	F: 5′-ATG ATT CGG GCC TTT GTT CAG CC-3′	324
		R: 5′-GGA ACT GAC GGT AGA GAT GGT TGC-3′	
exon1B	NM_001159533	F: 5′-ATG GTC AAT GAA AAC ACG AGG ATG-3′	234
		R: 5′-GGA ACT GAC GGT AGA GAT GGT TGC-3′	
exon8A	NM_009781	F: 5′-GCT GAC GGT GTT CCA GTG TA-3′	155
		R: 5′-TCA AAA CAC CGA GAA CCA GA-3′	
exon8B	NM_001290335	F: 5′-GCA TCA CCA ACT TCG ACA AC-3′	190
		R: 5′-GCT AAG AAC ACC GAG AAC CAA-3′	
exon9*-	NM_009781	F: 5′-ACC ATG GAG GGC TGG ACA GA-3′	339
exon9*+	NM_001290335	R: 5′-AGA CTC AGT CTC ACT TGT GGG-3′	414
exon31	NM_009781	F: 5′-GAG CAT AAT TGA TGT CAT TC-3′	136
		R: 5′-CTT CAC CAG GCG CAT GAC-3′	
exon32	NM_001255997	F: 5′-TGT TGA TAT AGC AAT CAC C-3′	130
		R: 5′-CTT CAC CAG GCG CAT GAC-3′	

F: forward primer – sense, R: reverse primer.

### Lymphatic vessel isolation

Mice were anesthetized with pentobarbital sodium (Nembutal; 60 mg kg^−1^, i.p.). An incision was made in one flank from the inguinal node to the axillary node to access the IALs, or on the dorsomedial side of either leg from the ankle to the groin to access the popliteal lymphatics (PLs)^52^. Rat mesenteric lymphatic vessels were excised from anesthetized rats as described previously^53^. The excised lymphatic vessel was pinned on a Sylgard platform (Sylgard® 184, Dow Corning, Midland, MI, USA), in Krebs’ buffer supplemented with 0.5% albumin, and subsequently isolated from the surrounding connective tissue and fat. After the surgery, the animals were euthanized with 200 mM KCl, i.c.

### Pressure myography

A segment of an excised lymphatic vessel containing at least one valve was transferred to a 3-mL chamber where it was cannulated onto two micropipettes and pressurized to 3 cmH_2_O. The bath solution containing Krebs buffer was exchanged at a rate of 0.5 mL/min and equilibrated for 30–60 minutes, at 37 °C, as previously described^52^. The inner diameter was tracked continuously from video images using a digital edge-detection method^54^. Vessels were included in the data analysis only if 1) spontaneous contractions and tone developed within the first 60 minutes; 2) normalized contraction amplitude exceeded 30% at 2 cmH_2_O intraluminal pressure (except for vessels from SMMHC-CreER^T2^; Cav1.2^fl/fl^ mice).

Each vessel was tested for contractile function using pressure steps between 0.5–8 cmH_2_O (for IALs) or 0.5–10 cmH_2_O (for popliteal lymphatics, PLs). After initial equilibration at P = 3 cmH_2_O, pressure was lowered to 2, 1, then 0.5 cmH_2_O, and then raised in steps from 0.5 to 10 cmH_2_O, while recording diameter for 2–3 min at each pressure. Contraction amplitude was typically optimal at P = 1 or 2 cmH_2_O while frequency increased with each pressure step from 0.5 to 5 cmH_2_O and reached a plateau at ~8 cmH_2_O. Tests using ACh, Bay K8644 or nifedipine were conducted at the pressure that produced the largest contraction amplitude (usually 2–3 cmH_2_O). To test a potential role for T-type VGCCs in lymphatic contractions, the inhibitors Ni^2+^ (15 µM-2 mM), nifedipine (1 nM–10 µM), mibefradil (1 nM–100 µM) or TTA-A2 (100 nM-100 µM) were added to the bath solution. Acetylcholine (ACh, 1–300 nM) was applied to test its ability to inhibit contractions through NO production^55^. The L-type VGCC activator Bay K8644 (1 µM) was added to test residual Ca_v_1.2 activity in vessels from tamoxifen-induced SMMHC-CreER^T2^; Ca_v_1.2^fl/fl^ mice. Inhibitors were applied in increasing concentrations to generate cumulative concentration-response curves. Contraction responses were recorded for 3 min before the next concentration was applied. At higher concentrations of some pharmacological inhibitors, the changes in inner diameter were <5 µm and were not entrained over the length of the vessel—i.e. they resembled the irregular vasomotion often observed in arteries; these changes were not counted as *bona fide* propulsive contractions^52^. At the end of every pressure myograph experiment the vessels were superfused for 30 min with Ca^2+^-free Krebs buffer solution containing 3 mM EGTA to obtain the passive diameter at each pressure.

### Calculation of contractile parameters

From internal diameter measurements, end diastolic diameter (EDD) and end systolic diameter (ESD) were determined for each contraction cycle, after which the following contraction parameters were calculated:

1 $${\rm{AMP}}={\rm{EDD}}-{\rm{ESD}}$$

2 $$ \% \,{\rm{Tone}}=[({\rm{MaxD}}-{\rm{EDD}})/{\rm{MaxD}}]\ast 100$$

3 $${\rm{Ejection}}\,{\rm{Fraction}}\,({\rm{EF}})=({{\rm{EDD}}}^{2}-{{\rm{ESD}}}^{2})/{{\rm{EDD}}}^{2}$$

4 $${\rm{Fractional}}\,{\rm{Pump}}\,{\rm{Flow}}\,({\rm{FPF}})={\rm{FREQ}}\ast {\rm{EF}}$$

where MaxD represents the maximum passive diameter obtained under Ca^2+^-free conditions at the same pressures at the end of the experiment. FREQ (min^−1^) was also calculated. For protocols using pharmacological inhibitors, the amplitude and frequency were sometimes normalized to the control values recorded prior to the first application of inhibitor as follows:

5 $${\rm{Normalized}}\,{\rm{AMP}}=({\rm{AMP}}/{\rm{MaxD}})\ast 100$$

6 $${\rm{Normalized}}\,{\rm{FREQ}}=({\rm{FREQ}}/{{\rm{FREQ}}}_{{\rm{avg}}})\ast 100$$

where FREQ_avg_ represents the average FREQ during the 2-minute baseline period prior to the addition of inhibitor.

### Sharp electrode recording of Vm

Mouse IALs lymphatic vessels were isolated and pressurized as described above. Once the vessel had stabilized at a pressure of 3 cmH_2_O, wortmannin was added (1–3 μM) and allowed to incubate for 30–60 minutes to inhibit myosin light chain kinase^56^ and blunt vessel movement associated with contractions to <5 μM, as assessed by edge-tracking of the vessel wall. Impalements into lymphatic smooth muscle were made using intracellular microelectrodes (300–350 MΩ) filled with 1 M KCl and membrane potential (Vm) was sampled at 1 KHz using a NPI SEC-05x amplifier (ALA instruments, Farmingdale, NY). Once impaled, Vm was allowed to settle for 15–30 seconds until the membrane potential stabilized. Vm was corrected for the offset value recorded upon withdrawal of the microelectrode from the cell. Acquired data files were then processed and action potentials (APs) were measured and characterized using an in-house Python algorithm described previously^52^. Mean total integrated signal per AP and percent-integrated signals for each phase of an AP were computed for each trace consisting of three or more APs per vessel per mouse.

### Single lymphatic smooth muscle cell isolation

After cleaning, collecting lymphatics were heated to 37 °C in low-Ca^2+^-digestion solution containing (in mM): 144 NaCl; 5.6 KCl; 0.1 CaCl_2_.2H_2_O; 1.0 MgSO_4_; 0.42 Na_2_HPO_4_.H_2_O; 0.44 NaH_2_PO_4_.H_2_O; 4.17 NaHCO_3_; 10 HEPES; 5 D-glucose (pH adjusted to 7.4 with NaOH, 25 °C) at 37 °C for 10 min, and then went through two digestion steps: (1) with 1 mg/mL dithioerythritol (Sigma, D8161–5G) and 1 mg/mL (mouse) papain (Sigma, P4762-1G) added to the low-Ca^2+^-digestion solution; (2) with 0.5 mg/mL collagenase H (Sigma, C8051-1G), 0.7 mg/mL collagenase F (Sigma, C7926-1G) and 1 mg/mL soybean trypsin inhibitor II (Sigma, T9128-1G) added to the low-Ca^2+^-digestion solution. After digestion, the remaining vessel fragments were rinsed with ice-cold digestion solution and triturated with a fire-polished Pasteur pipette to release single cells. Isolated muscle cells were stored in ice-cold solution to stop enzymatic digestion and used within 4–6 hr^21^. The resulting LMC cell homogenates were used either for Ca^2+^ current recording with the patch clamp technique or collection of single LMCs for end-point PCR. For LMC collection, the cell suspension was placed in Krebs containing 0.1 mM CaCl_2_ in a 0.5 mL chamber at room temperature and a 30 µm-pipette was used to collect single, spindle-shaped cells.

### Patch clamp recordings

LMCs isolated from the digestion of lymphatic vessels were placed in a recording chamber under an inverted microscope and perfused with physiological solution containing 1.8 mM Ca^2+^. Whole-cell patch clamp recording of current through VGCCs at room temperature was performed using an EPC9 amplifier (HEKA, Bellmore, NY, USA) controlled by Pulse software (HEKA) as described previously^57^). Recording electrodes (resistance = 3–5 MΩ) were back-filled with pipette solution containing Cs^+^ to block K^+^ current. Pipette movement was controlled with a hydraulic manipulator (MO-102, Narishige, Tokyo, Japan). After the gigaseal was formed and stabilized in physiological solution, all subsequent recordings were performed in bath solution containing 10 or 20 mM BaCl_2_ to enhance current through Ca_v_1.2/Ca_v_3×. Cell capacitance, an indication of cell membrane surface area, ranged from 9–25 pF. The recorded current value (pA) was normalized to cell capacitance (pF) for each cell to obtain current density (pA/pF). Holding potential was −70 mV. Two voltage clamp protocols were subsequently performed: (1) a voltage ramp, from −100 to +80 mV at 1 mV/ms, to rapidly obtain current-voltage relations; (2) voltage steps from a holding potential of −70 mV with a prepulse to −90 mV for 200 ms, followed by steps to a range of voltages from −80 to +40 mV (in 10 mV-increments, 2 s each), and a final step back to the holding potential; this protocol provided a measurement of current kinetics in response to different voltage steps^11^.

### Solutions and chemicals

Lymphatic vessels were isolated and cannulated in 0.5% albumin-supplemented Krebs buffer; the cannulating pipettes contained the same solution. Pressure myograph experiments were conducted in Krebs buffer without albumin. Krebs buffer contained (in mM): NaCl, 146.9; KCl, 4.7; CaCl_2_, 2; MgSO_4_, 1.2; NaH_2_PO4.H_2_O, 1.2; NaHCO_3_, 3; Na-HEPES, 1.5; D-glucose, 5 (pH 7.4, 37 °C). For patch clamp recordings, the physiological Ca^2+^ bath solution contained (in mM): 1.8 CaCl_2_, 110 NaCl, 1 CsCl, 1.2 MgCl_2_, 10 HEPES, 10 D-glucose (pH adjusted to 7.4 with NaOH). Ba^2+^ bath solution contained (in mM): 10 or 20 BaCl_2_, 110 NaCl, 1 CsCl, 1.2 MgCl_2_, 10 HEPES, 10 D-glucose (pH adjusted to 7.4 with NaOH). Patch pipettes contained (in mM): 135 CsCl, 10 HEPES, 10 EGTA, 5 GTP·Mg, (pH adjusted to 7.2 with CsOH). All chemicals were obtained from Sigma (St. Louis, MO, USA), except for BSA (US Biochemicals; Cleveland, OH, USA), MgSO_4_, HEPES (Fisher Scientific; Pittsburgh, PA, USA), and TTA-A2 (Alomone, Israel). TTA-A2, nifedipine and Bay K8644 were dissolved in DMSO. Ni^2+^, mibefradil and acetylcholine were dissolved in Krebs buffer (pre-warmed for Ni^2+^) without BSA. DMSO by itself, at its maximal concentration (0.033%) had no effect on contractions.

### End-point PCR

End-point PCR experiments detected message for VGCCs in mouse whole PLs and IALs and in LMCs freshly isolated from either of the two types of vessels. Vessels used for PCR were dissected at 4 °C. Cleaned lymphatic vessels or collected LMCs were directly transferred to RNAlater solution (Qiagen, Hilden, Germany) and stored at −80 °C. RNA was pooled from 100–300 LMCs collected by micropipette aspiration of spindle-shaped cells digested from 6–16 lymphatic vessels (from three or more mice). RNA was extracted using the Arcturus PicoPure RNA Isolation Kit (Life Technology, Carlsbad, CA, USA). cDNA then was synthesized using the Superscript III first Strand synthesis system (Invitrogen, Carlsbad, CA, USA). PCR was subsequently performed using GoTaq Flexi Reverse Transcriptase (Promega, Madison, WI, USA). Nested PCR was performed for weak signals such as Ca_v_3.1, Ca_v_3.2 and Ca_v_3.3. Nested PCR used a second internal set of primers to amplify the PCR product of the first external set of primers. The amplified PCR products were loaded onto 1.5–2% Agarose gel (Invitrogen) + Gel Red (1:10000, Biotum, Fremont, CA, USA) and the gel was run at 70–80 V for 60–90 min.

### Immunostaining

IALs were isolated and transiently pressurized to ~8 cmH_2_O and stretched axially to remove slack. The vessels were fixed in 2% paraformaldehyde (v/v) (19943-1 L, Affymetrix, Cleveland, OH, USA) for 30 minutes while pressurized to 3 cmH_2_O. The vessels were then washed in Phosphate Buffered Saline (PBS, 21600-010, Thermo Fisher Scientific, Waltham, MA, USA), and permeabilized with 0.3% Triton-X (v/v) (32737-1000, Acros Organics, Springfield Town, NJ, USA) for 30 minutes and blocked with 5% donkey serum (v/v) for 1 hr at room temperature. The vessels were incubated in α-actin primary antibody (Sigma, A2547) (1:200) and one of the primary antibodies for VGCCs (Alomone, Jerusalem, Israel): Ca_v_3.1 (#ACC-021), Ca_v_3.2 (#ACC-025) (1:100), Ca_v_1.2 (#ACC-003) diluted in PBS containing 5% donkey serum (017-000-121, Jackson ImmunoResearch Laboratories, West Grove, PA, USA), and 0.1% Triton-X (AC2156825000, Thermo Fisher Scientific). The vessels were washed with PBS at least 3 times for 20 min and incubated with an appropriate secondary antibody diluted 1:200 in PBS containing 5% donkey serum and 0.1% Triton-X for 1 h at room temperature. The secondary antibodies used were goat anti-mouse antibody Alexa Fluor 647 (A21241, Invitrogen, Carlsbad, CA, USA) and donkey anti-rabbit Alexa Fluor 448 (A21206, Invitrogen). The vessels were subsequently washed again with PBS at least 3 times for 20 min. Subsequently, ProLong® Gold Antifade Reagent with DAPI (P36931, Thermo Fisher Scientific) was used in some cases to stain nuclei and to mount the vessels onto imaging slides. The mounted vessels were covered with a #1 glass coverslip, allowed to cure overnight and sealed the following day with nail polish. Ca_v_1.2 staining served as a positive control and α-actin co-staining was used to outline the structure and location of LMCs. Negative controls included omission of Ca^2+^ channel primary antibody or primary antibody pre-absorption using the corresponding antigens (provided by Alomone). The vessels were recannulated and repressurized before collection of fluorescence images. Fluorescence emission was imaged using an Olympus IX-81 microscope equipped with an Andor/Yokogawa spinning disk confocal head and Hamamatsu Flash 4.0 camera (Hamamatsu Photonics, Shizuoka, Japan), with illumination from diode lasers (Andor, Belfast, Northern Ireland) using Metamorph (Nashville, TN, USA). Images were processed with NIH ImageJ.

### Data analysis

All data were analyzed using Prism 5 (Graphpad, La Jolla, CA, USA). Two-way ANOVAs with Bonferroni’s post hoc tests were performed to compare contractile function parameters between transgenic and WT vessels at different concentrations during concentration-response curve protocols. One-way ANOVAs were performed to compare pharmacological treatments with control parameters in the same vessels. P values less than 0.05 were considered statistically significant. The data are expressed as mean ± standard error of the mean.

## Results

### Effects of mibefradil and Ni^2+^ on rat mesenteric lymphatics

**Figure 1:**
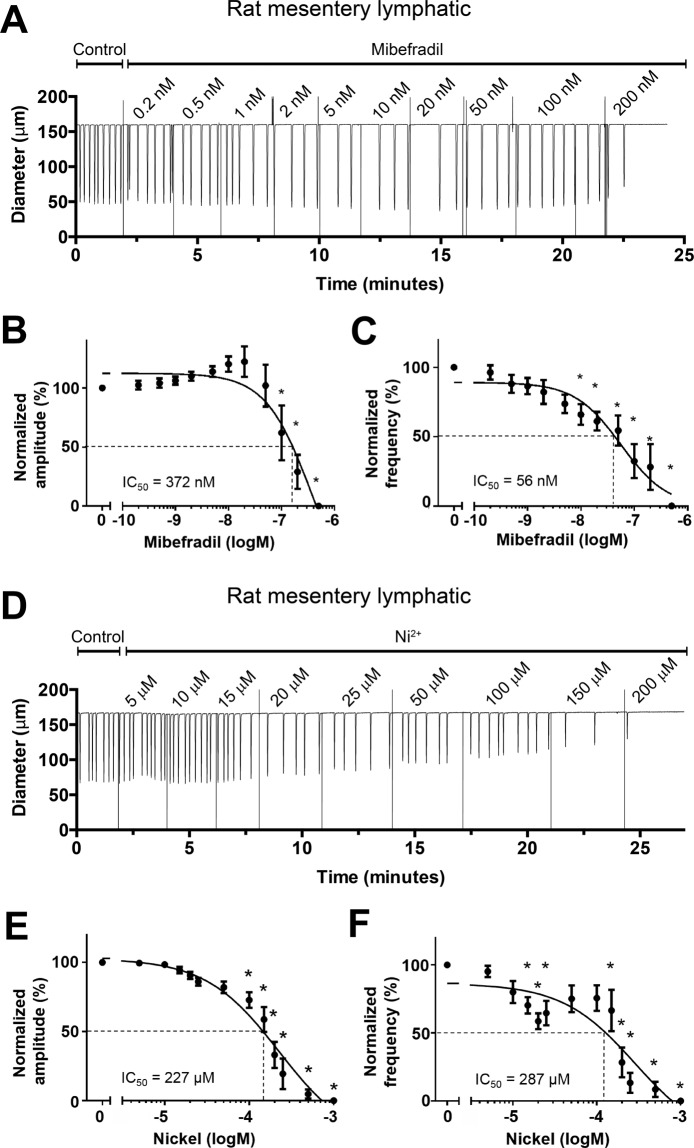
Responses of rat mesenteric lymphatics to mibefradil and Ni^2+^. Pressure = 3 cmH_2_O. Sample recording showing concentration-dependent effect of mibefradil on the contraction pattern. FREQ begins to slow at concentrations < 1 nM, with further slowing at concentrations up to 50 nM. At 200 nM, all activity ceased following a single contraction. (**B**) Summary of effects of mibefradil on AMP, showing tendency of AMP to rise at low mibefradil concentrations (also evident in **A**) before declining at concentrations > 50 nM. (**C**) Summary showing concentration-dependent inhibition of FREQ by mibefradil. The IC_50_ for FREQ was substantially lower than that for AMP, suggesting a selective inhibition of FREQ at low concentrations. (**D**) Sample recording showing concentration-dependent inhibition by Ni^2+^ of both AMP and FREQ of rat mesenteric lymphatics. (**E,F**) Summary of effects of Ni^2+^ on AMP (**E**) and FREQ (**F**). FREQ inhibition was biphasic, with partial inhibition at low concentrations, partial recovery at intermediate concentrations, followed by full inhibition at concentrations > 200 μM. The data in F could not be fit to the Hill equation and so the curve was interpolated and used to estimate an IC_50_ = 287 μM.

The goal of this study was to test the hypothesis that T-type VGCCs play an important role in lymphatic vessel pacemaking. Prior to performing experiments on mouse lymphatic vessels, we sought to confirm the expression of Ca_v_3.1 and Ca_v_3.2 in rat mesenteric LMC and the effects of the T-type VGCC inhibitors mibefradil and Ni^2+^ on rat mesenteric lymphatics as reported by Lee *et al*.^35^. To assess contractile parameters of lymphatic collectors under controlled conditions (fixed transmural pressure, absence of luminal flow), single-valve segments of rat mesenteric lymphatics were studied *ex vivo* using established pressure myograph methods^55^. All vessels used for further experimentation developed robust spontaneous contractions when pressurized to 3 cmH_2_O at 37 °C. With pressure maintained at that level, mibefradil was added to the bath in cumulative concentrations while assessing its effects on contraction amplitude and frequency for 2 min at each concentration. A representative recording of a spontaneously contracting rat mesenteric lymphatic during mibefradil application is shown in Fig. 1A. In this recording, Mibefradil slowed the contraction frequency, starting at concentrations below 1 nM and reaching a maximum effect at ~20 nM, but FREQ partially recovered at concentrations of 50 and 100 nM. Contraction amplitude was remarkably constant over the concentration range 1–100 nM, but spontaneous contractions stopped completely at 200 nM. The summary data in Fig. 1B,C reveals the same pattern of contractile regulation for 8 rat vessels, with a gradual reduction in FREQ occurring at all concentrations but only being significantly different from control at concentrations >10 nM. In contrast, there was a trend for AMP to increase slightly up to mibefradil concentrations of 100 nM, above which it fell precipitously. All vessels stopped contracting at the higher concentrations of mibefradil and the large error bars for the points at concentrations between 50–200 nM reflect the fact that some vessels stopped contracting at slightly different concentrations than others. The IC_50_ of mibefradil for AMP was 372 nM and the IC_50_ for FREQ was 56 nM. The lower IC_50_ for FREQ is consistent with the results of Lee *et al*.^35^, who concluded that mibefradil selectively inhibited FREQ, but not AMP, of rat mesenteric lymphatics, although those authors tested only a single concentration (100 μM) of mibefradil.

Complete concentration-response curves for Ni^2+^ were also performed on rat mesenteric lymphatics. With pressure maintained at 3 cmH_2_O, Ni^2+^ was added to the bath in increasing concentrations while recording its effects on contraction AMP and FREQ for 2 min at each concentration. A representative recording of a rat mesenteric lymphatic responding to Ni^2+^ is shown in Fig. 1D. FREQ began to slow when Ni^2+^ reached a concentration of 15 μM and slowed further at concentrations of 20 and 25 μM, then increased somewhat at 50 and 100 μM before slowing further at 150 µM and stopping completely at 200 μM. In contrast, AMP declined progressively throughout the entire concentration range of Ni^2+^, beginning at ~15 μM. The summary data for 8 vessels in Fig. 1E,F showed a similar response pattern: a progressive reduction in AMP with increasing concentrations of Ni^2+^, becoming significantly different from control above concentrations of 50 μM. FREQ exhibited a biphasic concentration-response relationship to Ni^2+^, with significant inhibition at concentrations between 10–40 μM, a rebound at concentrations between 50–105 μM followed by further inhibition and complete cessation of contractions at concentrations >200 μM. The IC_50_ for Ni^2+^ on AMP was 227 μM and, although the FREQ data could not be fit to the Hill equation, the interpolated IC_50_ for Ni^2+^ on FREQ was 287 μM. These results are somewhat, but not completely, consistent with those of Lee *et al*.^35^. The inhibition of FREQ by low concentrations of Ni^2+^ could be explained by inhibition of Ca_v_3.2 channels^23,33^; however, the complete inhibition of contraction by both drugs at intermediate-to-high concentrations suggests off-target effects on L-type VGCCs, which would be consistent with previous reports^14,58^. What is not clear is the extent to which either or both, Ni^2+^ and/or mibefradil, might have inhibited L-type VGCCs at lower concentrations. These uncertainties point to the need for using a different method to inhibit T-type VGCCs, which is the reason we turned to genetic approaches in the mouse.

### VGCC isoform expression in mouse lymphatics

**Figure 2:**
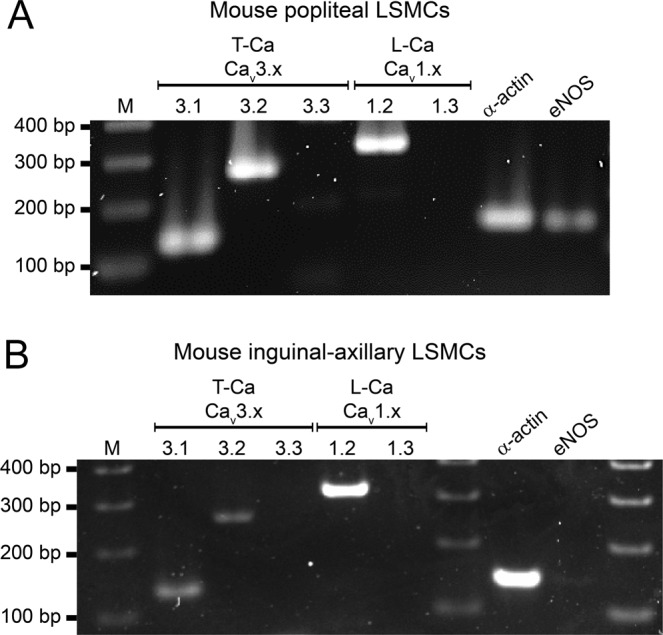
PCR to detect VGCC isoforms in mouse LMCs isolated from popliteal (**A**) or IALS (**B**). LMCs were collected by micropipette aspiration after isolation, cleaning and digestion of the respective vessels. Ca_v_3.1 and Ca_v_3.2, but not Ca_v_3.3 isoforms are detected in LMCs from both vessels. Ca_v_1.2 shows a strong band in both samples. eNOS is detected in cells collected from PLs (**A**) indicating that there is some contamination by endothelial cells (see Suppl. Fig. 1 for additional information). M = marker; bp = base pairs.

Before testing the possible role of T-type VGCCs in the spontaneous contractions of mouse lymphatics, we examined which Ca_v_3 isoforms were expressed in mouse lymphatic vessels. We tested two types of collecting lymphatic vessels from different regions of the mouse, based on the following reasoning. Popliteal afferent lymphatics (PLs) previously have been demonstrated by our laboratory^55^ and others^59^ to exhibit robust spontaneous contractions *ex vivo* comparable in magnitude to those from other species^60^; further, these contractions are modulated by pressure in the same way, and over approximately the same range, as those of collecting lymphatic vessels from other species, in particular rat mesentery^61^. In contrast, the IAL is an efferent vessel that demonstrates strong spontaneous contractions^62^ but is larger, easier to clean and cannulate, and more amenable to electrophysiology studies. PL and IAL vessels were excised from WT (C57BL/6J) mice and thoroughly cleaned of fat and connective tissue. RNA was extracted and end-point PCR performed on single (whole) vessels, 2–3 mm in length. Message for Ca_v_1.2 was detected in both types of vessels. Message for Ca_v_3.1 and Ca_v_3.2 was detected in PLs, and message for all three Ca_v_3 isoforms was detected in IALs (Suppl. Fig. S1). However, Ca_v_3.3 was not detected by immunostaining (Suppl. Fig. S11). Because analysis of mRNA obtained from whole lymphatic vessels includes mRNA from multiple cell types, including LMCs, LECs, dendritic cells^63,64^, macrophages, mast cells^65,66^, lymphocytes, and possibly neuronal axons and terminals, we could not be confident which VGCC isoforms are actually expressed by LMCs per se. Therefore, we repeated the procedure with the additional step of enzymatically digesting the vessels with papain and collagenase followed by vigorous trituration in a fire-polished Pasteur pipette to release single cells. Spindle-shaped cells, presumably representing LMCs, were then collected into a 20-μm tip micropipette using suction from a micrometer-controlled glass syringe. The collected cells (~100–300 per animal) were deposited into a clean glass chamber, pooled, RNA was extracted, and PCR performed with the same primer sets used above. LMCs from both vessel types expressed message for Ca_v_3.1, Ca_v_3.2 and Ca_v_1.2 (Fig. 2). The absence of Ca_v_3.3 in both purified LMC preparations suggests that it was contributed by other cell types in the initial whole-vessel PCR analyses. The PL sample continued to show a band for eNOS, suggesting some contamination from LECs; however, the IAL sample did not. Because previous studies suggest that T-type VGCCs might be expressed in endothelium^67^, we checked for Ca_v_3 expression specifically in LECs by performing additional PCR analyses on LEC tubes isolated from PLs. Vessels were dissected and cleaned but then subjected briefly to collagenase (alone) treatment and gentle trituration using a micropipette with a tip diameter just larger than that of the vessel. This procedure has been used consistently to isolate pure EC tubes from arterioles^68^ and we adapted it recently for lymphatic vessels^69^). Analysis of LEC tubes obtained from PLs showed a prominent band for eNOS, no band for SMA and no message for Ca_v_3.1 or Ca_v_3.2 (Suppl. Fig. S1D**)**. The LEC tube sample failed to show message for Ca_v_1.2 (not shown).

**Figure 3:**
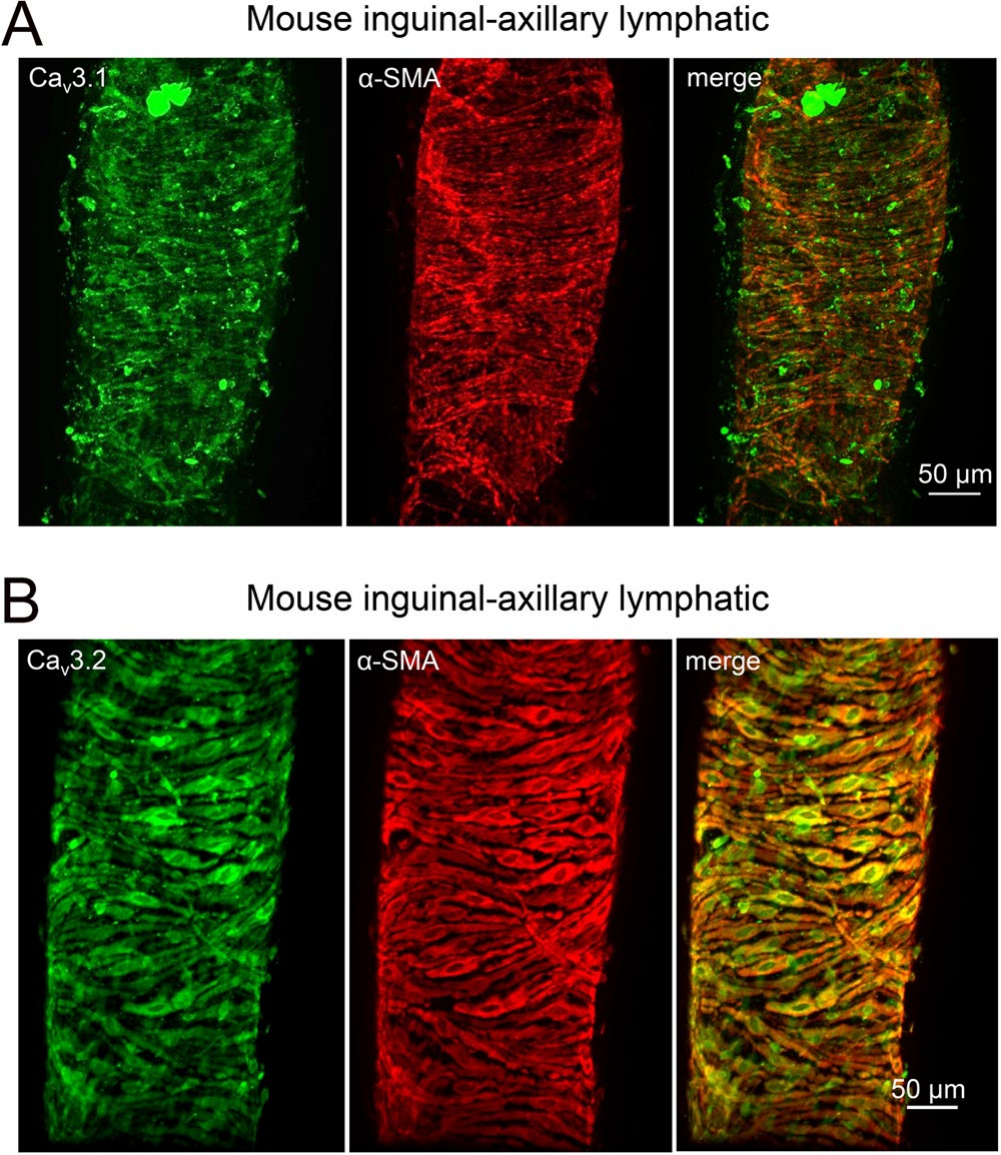
Immunostaining of mouse IALs for (**A**) Ca_v_3.1 or (**B**) Ca_v_3.2. Both samples were co-stained for SMA (smooth muscle α-actin) and the green (Ca_v_3) channels and red (SMA) channels showed substantial overlap, suggesting co-localization of both isoforms (particularly Ca_v_3.2) with smooth muscle cells. The right panels show the merged channels.

Expression levels of Ca_v_3 isoforms were too low and sample sizes too small to make Western blotting feasible. Therefore, to test for protein expression, we performed immunostaining for Ca_v_3.1 and Ca_v_3.2 using IALs from WT mice. Both Ca_v_3 isoforms showed prominent signals in the green fluorescence channel for the secondary antibody that appeared to substantially colocalize with the signal for SMA in the red channel (Fig. 3), albeit with some non-specific staining. For negative controls, WT vessels were stained using the same primary and secondary antibodies and image acquisition/analysis settings after preabsorption with the respective antigenizing peptides (Suppl. Figs. S2 and S3); only non-specific background signal, which did not colocalize with SMA, was detected in each type of vessel. As additional controls, we stained IALs from Ca_v_3.1^−/−^ mice for Ca_v_3.1 and IALs from Ca_v_3.2^−/−^ mice for Ca_v_3.2 and only non-specific background signal was noted in the green channel for each (Suppl. Figs. S2 and S3). Mesenteric arterioles, which are known to express both Ca_v_3 isoforms^70,71^, served as positive controls for each antibody (Suppl. Figs. S2 and S3), and expressed strong signal for both Ca_v_3.1 and Ca_v_3.2 that colocalized with that for SMA. Collectively, these analyses suggest that message and protein for both Ca_v_3.1 and Ca_v_3.2, but not Ca_v_3.3, are expressed in the LMC layer of mouse lymphatic vessels.

### Tests for functional Ca_v_3 channels in mouse LMCs

**Figure 4:**
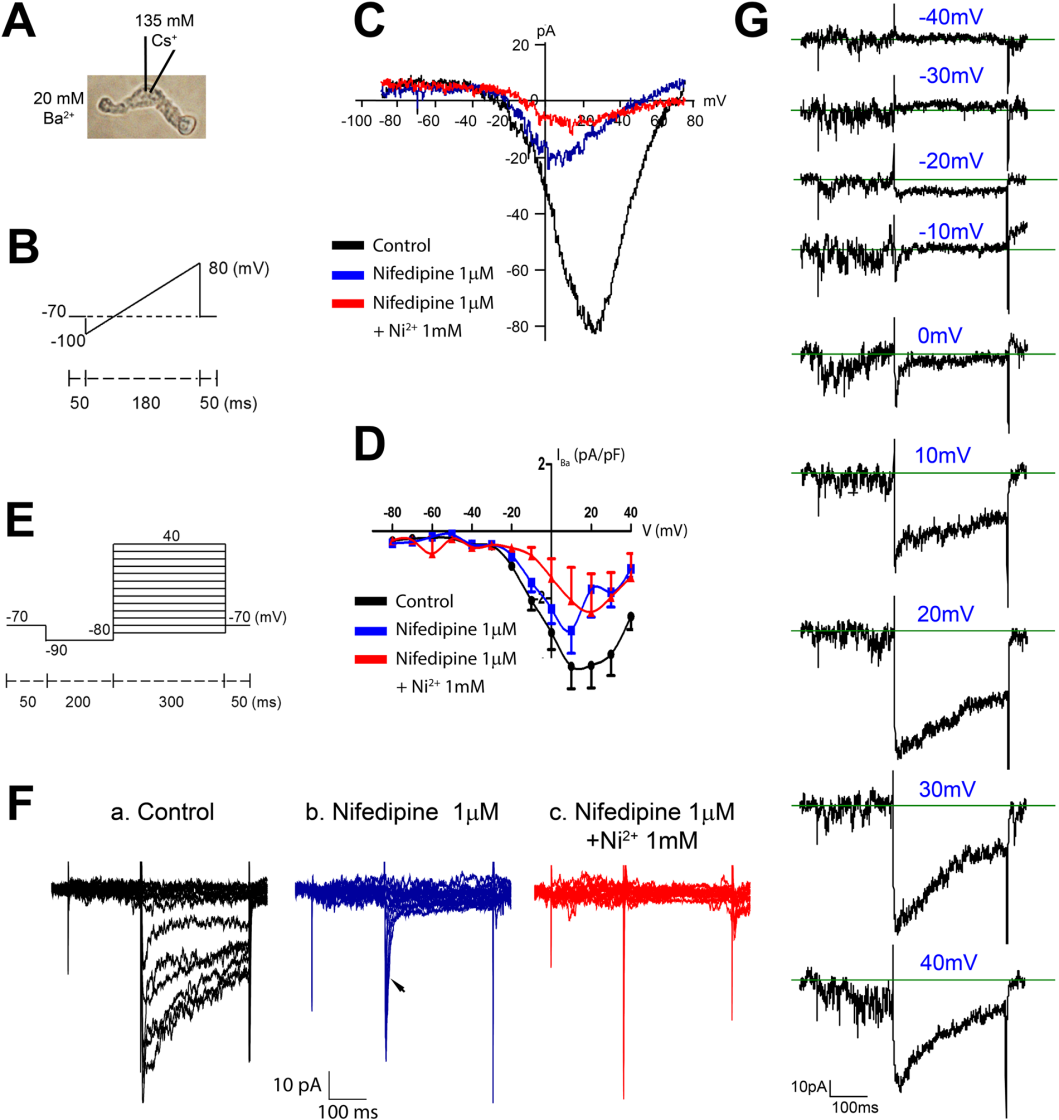
Whole-cell patch clamp recordings of VGCC current in LMCs isolated from WT mouse IALs. (**A**) Brightfield image of an isolated LMC with the major components of the pipette and bath solutions indicated. (**B**) Ramp protocol to estimate I-V relationships. (**C**) Example I-V curve for an LMC under control conditions (black), after addition of 1 μM nifedipine (blue), which blocked ~80% of the current and shifted the peak from +20 to 0 mV, and after addition of 1 μM nifedipine + 1 mM Ni^2+^ (red), which blocked almost all of the remaining current. (**D**) Summary I-V curves for 5 LMCs isolated from mouse IALs studied with the protocol shown in (**B**,**C**). (**E**) Voltage step protocol. (**F**) Example recordings from a LMC using the voltage step protocol, showing (**a**) the initial whole-cell current, (**b**) inhibition of the long-lasting component of the inward current by nifedipine but preservation of a transient inward current (arrow), and (**c**) elimination of all inward current in 1 μM nifedipine + 1 mM Ni^2+^ (the three remaining spikes are capacitance artifacts and are also present in the other recordings). (**G**) The same traces as in panel Fa except stacked vertically to show the small transient inward currents that activate at the beginning of the steps to −10 and 0 mV (and are then masked by the longer lasting components at more positive voltage steps).

To test for the expression of functional Ca_v_3 channels, we enzymatically isolated LMCs from IALs or PLs and performed whole-cell patch clamp experiments using solutions and conditions designed to maximize VGCC current. 20 mM Ba^2+^ or Ca^2+^ was used as the charge carrier in the bath solution; outward K^+^ currents, that otherwise would have masked smaller currents through VGCCs, were blocked by dialyzing the cells with 135 mM Cs^+^ solution. After enzyme treatment and trituration, most LMCs were easily distinguished as spindle shaped cells, with slightly rounded ends (Fig. 4A), but only a minority of such cells produced recordings with measurable (>20 pA per cell) inward divalent cation current. We confirmed the spindle-shape morphology of LMCs by using the same isolation procedure on lymphatic vessels from SMMHC^GFP^ mice and verifying the typical spindle shape of a GFP + cell. However, isolation of cells from Prox1^GFP^ vessels also yielded LECs that were spindle-shaped, perhaps explaining the low success rate in finding cells with measurable inward divalent cation current and accounting for residual eNOS message in spindle-shaped cells collected from popliteal lymphatics via micropipette aspiration for PCR (Fig. 2A).

The first patch clamp protocol was designed to evoke the opening of VGCCs using a voltage ramp from −100 to +80 mV from a holding potential of −70 mV (Fig. 4B). A hyperpolarizing step to −100 mV was expected to reset T-type VGCCs, if present, so they could contribute to inward current during the depolarizing ramp. As shown in the example in Fig. 4C, the (current-voltage) I–V curve produced by this protocol in a LMC isolated from a mouse IAL shows inward current activating at ~−20 mV and peaking at ~+20 mV. Such curves are expected to be right-shifted by 20–30 mV due to both the use of Ba^2+^ as the charge carrier and the use of a concentration that is higher than physiological levels (2 mM^72^). Attempted recordings in 2 mM Ca^2+^ bath solution typically produced inward currents that were <5 pA; for this reason, we were unable to determine the I-V curve in physiological Ca^2+^ solution, which would have been useful for assessing the window currents for T- (and L-) type VGCCs. The application of nifedipine during the recording in Fig. 4C reduced peak inward current from −80 pA to −20 pA and shifted the peak from +22 mV to +5 mV; this shift is consistent with unmasking of a T-type VGCCC current^21,70^. In this cell, the remaining (nifedipine-insensitive) current was blocked ~60% by the addition of 1 mM Ni^2+^ (lower Ni^2+^ concentrations produced less block), leaving some residual current for which the peak shifted right-ward to a more positive voltage; the residual current may reflect the presence of another Ca_v_ isoform (although it is unlikely to be Ca_v_1.3, see RT-PCR in Suppl. Fig. S1A–C) or other Ca^2+^-permeable cation channel(s). The I-V curve for 5 LMCs from IALs is summarized in Fig. 4D and shows the same pattern as described for the recording in panel C. Recordings from LMCs isolated from mouse popliteal lymphatics and from rat mesenteric lymphatics also showed the same type of inward currents with similar I-V relationships and nifedipine and Ni^2+^ sensitivities, but with smaller whole-cell currents (Suppl. Fig. S4A,B).

Because some inactivation of VGCCs is likely to occur during a voltage ramp, we also used voltage step protocols, as depicted in Fig. 4E, stepping first to −90 mV to relieve inactivation, followed by depolarizing steps from −80 to +40 in 10 mV increments in successive sweeps. Fig. 4Fa shows a typical recording, with each of the resultant current traces overlaid. Comparison of the control traces (in the absence of inhibitor) to those with 1 μM nifedipine (Fig. 4Fb) revealed a transient, rapidly-inactivating inward current that was nifedipine-insensitive (arrow); in this cell the transient current was completely blocked by 1 mM Ni^2+^ (Fig. 4Fc). The individual current traces for the same cell are aligned vertically in Fig. 4G to reveal that the transient current begins to activate at −20 mV, is the only inward current activated at −10 and 0 mV, and then is masked by a longer-lasting, slowly-inactivating inward current at more positive step potentials. These transient currents were not blocked by TTX (not shown). Collectively, the kinetics and nifedipine-resistance of this current are consistent with those of Ca_v_3 channels, in agreement with a preliminary report by Hollywood *et al*.^73^ using sheep LMCs.

### Effects of Ni^2+^ and TTA-A2 on mouse lymphatic contractions

**Figure 5:**
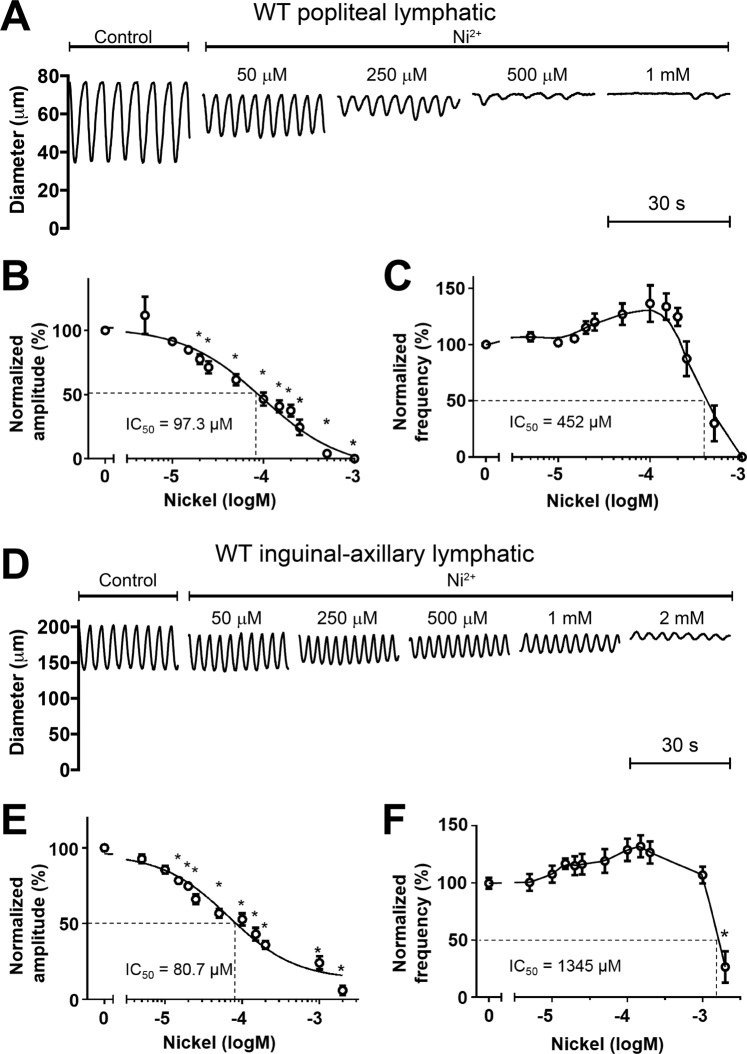
Responses of PLs and IALs from WT mice to Ni^2+^. (**A**) Example recording of spontaneous contractions in a PL before and after 4 different concentrations of Ni^2+^, showing a slight increase in FREQ at the two lowest concentrations compared to progressive reduction in AMP at all four concentrations. (**B,C**) Summary of contraction data for 8 PLs in response to 12 concentrations of Ni^2+^, given cumulatively. The increase in FREQ at concentrations below 0.3 mM is also evident in the graph in (**C**). (**D**) Example recording of spontaneous contractions of an IAL before and after 5 different concentrations of Ni^2+^. (**E,F**) Summary data for 8 IALs in response to 11 different concentrations of Ni^2+^. Again, there is a tendency for FREQ to increase, or be unaffected at concentrations below 1 mM, before declining substantially at 2 mM.

Having established that functional T-channels are present in mouse lymphatic smooth muscle, we then tested the effects of Ni^2+^ on the spontaneous contractions of PLs and IALs. A representative recording of spontaneous contractions in a PL during the application of increasing concentrations of Ni^2+^ is shown in Fig. 5A. Ni^2+^ produced a concentration-dependent reduction in AMP at all concentrations, but a slight increase in FREQ at concentrations up to 250 μM and then a reduction in FREQ at 500 μM before inhibiting contractions completely at 1 mM. Summary data for Ni^2+^ on PL contractions showed the same patterns (Fig. 5B,C). Interestingly, the IC_50_ for Ni^2+^ on AMP was 97 μM but on FREQ was 452 μM, which is opposite to the predictions and conclusions of Beckett *et al*.^8^ and Lee *et al*.^35^. A representative concentration-response curve for an IAL is shown in Fig. 5D. Increasing concentrations of Ni^2+^ led to progressive reductions in AMP and increases in FREQ until at 1 mM, spontaneous contractions ceased completely for ~45 sec, followed by partial recovery. Summary data for Ni^2+^ on 8 IALs showed the same pattern: a progressive reduction in AMP with a concomitant increase in FREQ until contractions stopped at a concentration of 2 mM (Fig. 5E,F). We also tested a recently developed and reputedly more selective inhibitor of Ca_v_3 channels, TTA-A2, which has been reported to be effective on Ca_v_3 channels at concentrations of 1 µM^74^. However, TTA-A2 had only a modest effect on both PLs and IALs, inhibiting AMP by ~20% at a concentration of 100 μM and having no significant effect of FREQ at any concentration (Suppl. Fig. S5). IC_50_ values for Ni^2+^, mibefradil and TTA-A2 on the various lymphatic preparations are listed in Table 2. In summary, the inconsistent effects of mibefradil and Ni^2+^ on AMP and FREQ of rat mesenteric lymphatics and of Ni^2+^ and TTA-A2 on mouse PLs and IALs highlight the importance of using more selective methods, e.g. genetic approaches, to assess the role(s) T-type VGCCs in lymphatic contraction.

**Table 2:** Summary of IC_50_s for pharmacological inhibitors of T-type VGCCs on lymphatic contraction amplitude and frequency.

Drug	Species	LV location	IC_50_ for amplitude	IC_50_ for frequency
Mibefradil	Rat	Mesenteric	66 nM	423 nM
Ni^2+^	Rat	Mesenteric	248 µM	279 µM
	Mouse	IAL	66 µM	1287 µM
	Mouse	Popliteal	110 µM	452 µM
TTA-A2	Mouse	Popliteal	1.3 µM	nd

nd = not-determined.

### Contractile and ACh responses of lymphatics from Ca_v_3.2^−/−^ mice

**Figure 6:**
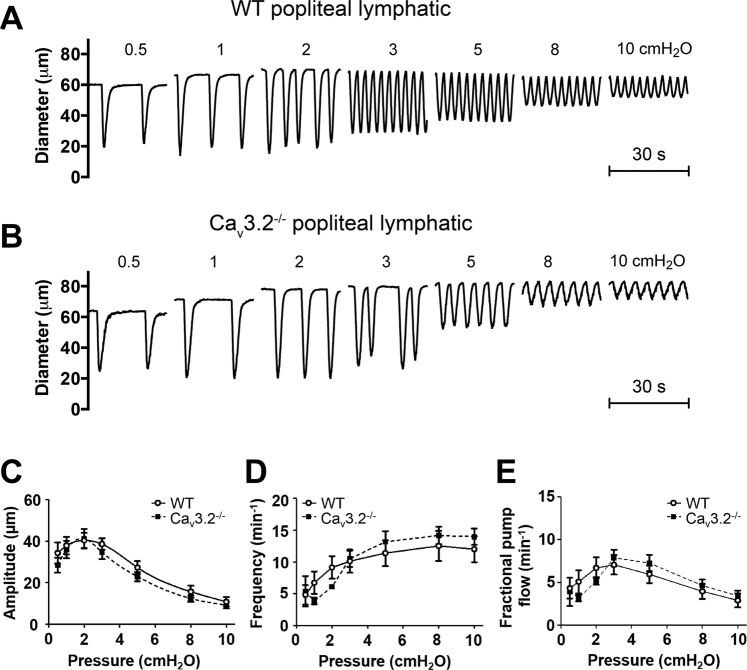
Comparison of contractile patterns and responses to pressure of PLs from WT and Ca_v_3.2^−/−^ mice. (**A,B**) Example recordings of spontaneous contractions at 7 different pressures in a WT vessel (**A**) and a Ca_v_3.2^−/−^ vessel (**B**). (**C–E**) Summary of comparisons of AMP, FREQ and FPF between WT and Ca_v_3.2^−/−^ PLs at 6 different pressures. None of the parameters were significantly different between the two groups at any pressure.

**Figure 7:**
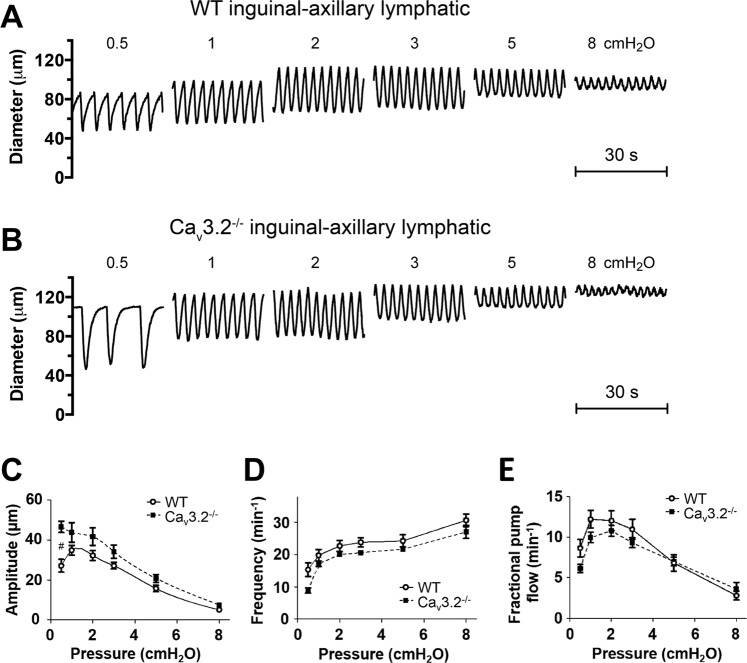
Comparison of contractile patterns and responses to pressure of IALs from WT and Ca_v_3.2^−/−^ mice. (**A,B**) Example recordings of spontaneous contractions at 7 different pressures in a WT vessel (**A**) and a Ca_v_3.2^−/−^ vessel (**B**). (**C–E**) Summary of comparisons of AMP, FREQ and FPF between WT and Ca_v_3.2^−/−^ IALs at 6 different pressures. None of the parameters were significantly different between the two groups at any pressure, except for AMP at 0.5 cmH_2_O, where the Ca_v_3.2^−/−^ group was higher.

We first tested vessels from Ca_v_3.2^−/−^ mice because the data in Fig. 1F, showing impairment of FREQ of rat mesenteric lymphatics at low concentrations (10–20 μM) of Ni^2+^, are consistent with the possible inhibition of, and role for, Ca_v_3.2 channels^23,33^. Representative recordings of PL diameter over time from WT and Ca_v_3.2^−/−^ mice are shown in Fig. 6A,B. The contraction patterns of the Ca_v_3.2^−/−^ vessel were quite similar to those of WT vessels at all pressures— in both the absolute values of AMP and FREQ as well as the normalized changes in AMP and FREQ with pressure. The data for PLs from 8 WT and 6 Ca_v_3.2^−/−^ mice are summarized in Fig. 6C–E. This summary analysis confirmed a lack of consistent, significant differences in AMP, FREQ or calculated FPF between WT and Ca_v_3.2^−/−^ PLs. A similar analysis is shown for IALs from WT and Ca_v_3.2^−/−^ mice in Fig. 7. Again, there were no significant differences between vessels from the two genotypes, except that AMP was higher in Ca_v_3.2^−/−^ IALs at a single pressure, 0.5 cmH_2_O (Fig. 7C). Additional contractile data for each of the two genotypes (EDD, EF, tone) are shown in Suppl. Fig. S6 and also reveal no significant differences for either vessel type compared to their respective control vessels. Because the penetrance of some transgenic traits can be strain-specific, we obtained an additional strain of Ca_v_3.2^−/−^ mice on a mixed background (mostly C129SVE) from the MMRRC (Columbia, MO) and tested the responses of six PLs to pressure. The representative sample traces and summary data show that the contraction patterns were nearly identical to those of WT PLs and Ca_v_3.2^−/−^ PLs on the C57BL/6J background (Suppl Fig. S7A–C).

Because Ca_v_3.2 activation has been shown to be linked to calcium spark activity, smooth muscle hyperpolarization and negative feedback inhibition of tone in cerebral and mesenteric arteries^23,33^, we tested whether the known effects of vasodilation and inhibition of lymphatic pumping produced by ACh would be altered in Ca_v_3.2^−/−^ lymphatics. PLs were isolated, cannulated and pressurized to 3 cmH_2_O. After spontaneous contractions developed, ACh was added to the bath in cumulative concentrations. ACh caused a concentration-dependent reduction in both AMP and FREQ of WT PLs (Suppl. Fig. S8A), typically beginning at 1–3 nM, which is consistent with previously published data^55^. Nearly identical inhibitory responses were observed for PLs from Ca_v_3.2^−/−^ mice (Suppl. Fig. S8B). Summary data are shown in Suppl. Fig. S8C–F, where FREQ is expressed both in absolute values and normalized to the respective control values in the absence of ACh. No significant differences were evident between the two data sets nor for the additional Ca_v_3.2 KO strain (Suppl. Fig. 7D–F). The trend for FREQ and normalized FREQ to increase at the highest concentration (Suppl Fig. 8B) reflects a stimulatory action of ACh^55^ on the smooth muscle layer of some vessels (even higher concentrations would have stimulated all vessels). Collectively, these results strongly suggest that Ca_v_3.2 does not play a detectable role in the regulation of either the FREQ or AMP of spontaneous contractions by pressure, or for ACh-mediated inhibition of lymphatic contractions in mouse PLs and IALs.

### Contractile and ACh responses of lymphatics from Ca_v_3.1^−/−^ mice

**Figure 8:**
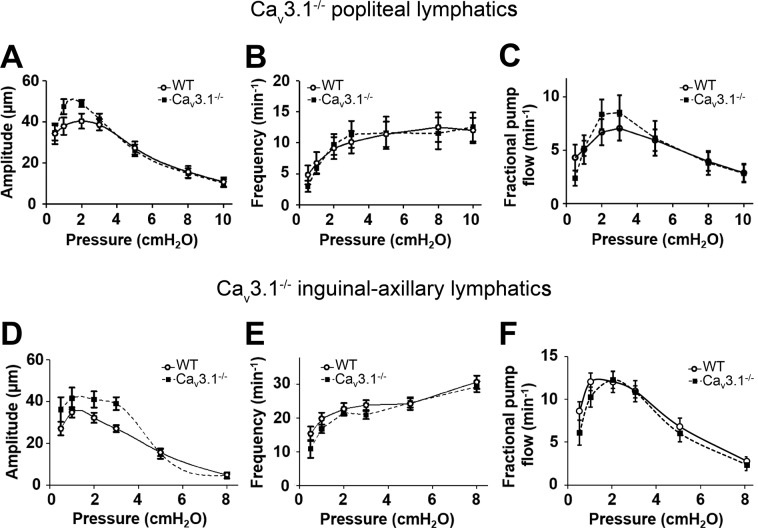
Comparison of responses to pressure of PLs and IALs from WT and Ca_v_3.1^−/−^ mice. (**A–C**) Summary of comparisons of AMP, FREQ and FPF between WT and Ca_v_3.1^−/−^ PLs at the 7 different pressures. None of the parameters were significantly different between the two groups at any pressure. (**D–F**) Summary of comparisons of AMP, FREQ and FPF between WT and Ca_v_3.1^−/−^ IALs at the 6 different pressures. None of the parameters were significantly different between the two groups at any pressure.

We then tested the hypothesis that Ca_v_3.1 channels play a critical role in regulating the FREQ of spontaneous lymphatic contractions. PLs and IALs were isolated from WT and Ca_v_3.1^−/−^ mice and contractile parameters were assessed at pressures from 0.5 to 10 cmH_2_O. The data for PLs are summarized in Fig. 8A–C and the data for IALs are summarized in Fig. 8D–F. There were no significant differences in AMP, FREQ or FPF at any pressure between WT and Ca_v_3.1^−/−^ vessels. These data are inconsistent with a critical contribution of Ca_v_3.1 channels to the FREQ or AMP of spontaneous contractions. We also tested ACh responses of PLs from Ca_v_3.1^−/−^ mice because that isoform in smooth muscle has been implicated as a target of endothelium-derived NO^21,75^; the results are shown in Suppl. Fig. S9. There were no significant differences between the two groups in the effectiveness of ACh to inhibit spontaneous contractions, although there was a trend for the AMP of Ca_v_3.1^−/−^ vessels to be more resistant to ACh (Suppl. Fig. S9C). Collectively, the results in Fig. 8 and Suppl Fig. 9 contradict the hypothesis that Ca_v_3.1 channels are important regulators of FREQ, but not AMP, of spontaneous lymphatic contractions, or that Ca_v_3.1 channels are important for mediating ACh responses of mouse PLs or IALs.

### Contractile and ACh responses of lymphatics from Ca_v_3.1^−/−^; Ca_v_3.2^−/−^ double KO mice

**Figure 9:**
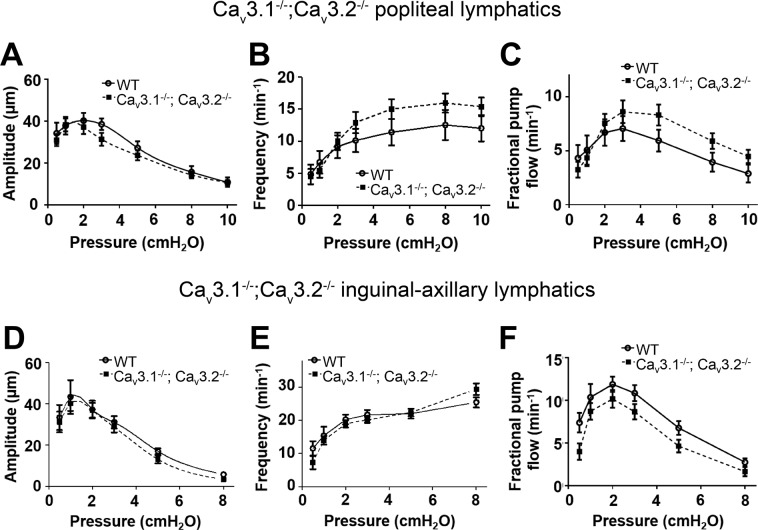
Comparison of responses to pressure of PLs and IALs from WT and Ca_v_3.1^−/−^; Ca_v_3.2^−/−^ double KO mice. (**A–C**) Summary of comparisons of AMP, FREQ and FPF between WT and Ca_v_3.1^−/−^; Ca_v_3.2^−/−^ PLs at the 7 different pressures. None of the parameters were significantly different between the two groups at any pressure. (**D–F**) Summary of comparisons of AMP, FREQ and FPF between WT and Ca_v_3.1^−/−^; Ca_v_3.2^−/−^ IALs at the 6 different pressures. None of the parameters were significantly different between the two groups at any pressure.

In some knock-out mouse models, deletion of one protein isoform is known to be compensated by upregulation of another isoform of that protein^76–79^. Compensation becomes more likely in global knock-out animals because it can occur gradually during development. We considered the possibility that deletion of Ca_v_3.2 could be compensated by upregulation of Ca_v_3.1 and vice-versa. Therefore, we generated mice deficient in both Ca_v_3 isoforms by breeding Ca_v_3.2^−/−^ mice with Ca_v_3.1^−/−^ mice to obtain offspring deficient in both isoforms. PLs and IALs from Ca_v_3.1^−/−^; Ca_v_3.2^−/−^ double KO mice were then studied at pressures from 0.5 to 10 cmH_2_O and the results are summarized in Fig. 9. Again, there were no significant differences in AMP, FREQ or FPF between Ca_v_3.1^−/−^; Ca_v_3.2^−/−^ double KO vessels compared to WT controls for either PLs or IALs. We then tested the ACh responses of PLs from Ca_v_3.1^−/−^; Ca_v_3.2^−/−^ mice and found no significant or consistent differences between the two groups at any ACh concentration (Suppl Fig. S10). Collectively, these results contradict the hypothesis that either Ca_v_3.1 or Ca_v_3.2 channels are critical regulators of spontaneous lymphatic contractions or ACh-mediated contraction inhibition.

### Effects of Ni^2+^ and mibefradil on contractile responses of popliteal lymphatics from WT and Ca_v_3.1^−/−^; Ca_v_3.2^−/−^ double KO mice

**Figure 10:**
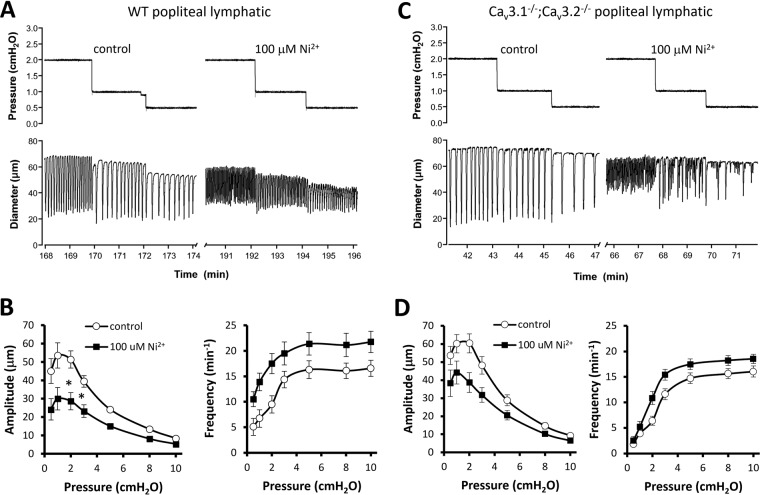
Effects of 100 μM Ni^2+^ on contraction amplitude and frequency over the entire pressure range for popliteal lymphatics from WT and Ca_v_3.1^−/−^; Ca_v_3.2^−/−^ double KO mice. (**A**) Example recordings from a WT popliteal lymphatic before and after addition of 100 μM Ni^2+^ to the bath. (**B**) Summary data for spontaneous contraction amplitude and frequency from eight WT vessels. 100 μM Ni^2+^ reduced amplitude by 40–50% at pressures < 3 cmH_2_O and by 20–30% at higher pressures. Ni^2+^ caused an increase in frequency at all pressures, with a more pronounced effect at lower pressures. (**C**) Example recordings from a Ca_v_3.1^−/−^; Ca_v_3.2^−/−^ double KO mouse before and after addition of 100 μM Ni^2+^ to the bath. Summary data for amplitude and frequency at each pressure are shown in D for nine Ca_v_3.1^−/−^; Ca_v_3.2^−/−^ vessels. Ni^2+^ led to 20–30% reduction in amplitude at all pressures and an increase in frequency at all pressures except 0.5 cmH_2_O. At the two lowest pressures contraction amplitude was highly variable over time, with small contractions interspersed with intermediate and large-amplitude contractions. For this analysis we did not count contractions less than 5 μm in amplitude at pressures ≤ 3 cmH_2_O because they were non-propulsive and usually not highly entrained. Counting these contractions would have led to even higher determinations of frequency at those pressures.

**Figure 11:**
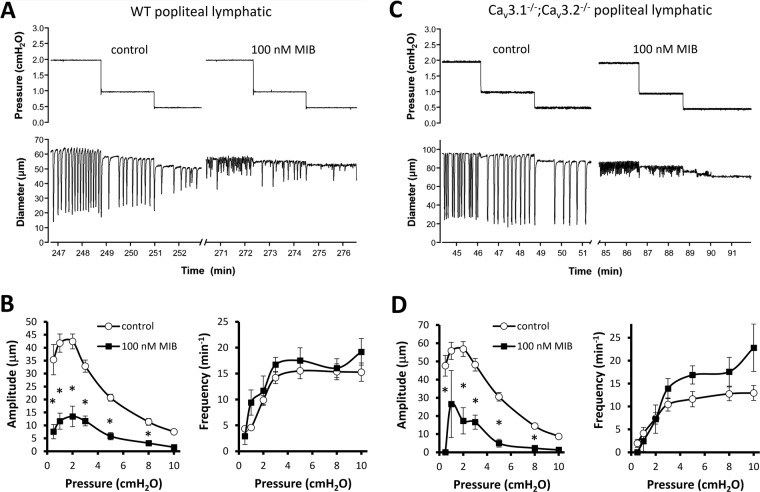
Effects of 100 nM mibefradil on contraction amplitude and frequency over the entire pressure range for popliteal lymphatics from WT and Ca_v_3.1^−/−^; Ca_v_3.2^−/−^ double KO mice. (**A**) Example recordings from a WT popliteal lymphatic before and after addition of 100 nM mibefradil to the bath. Mibefradil severely depressed contraction amplitude at all pressures and slightly increased frequency at almost all pressures (except 0.5 cmH_2_O). (**B**) Summary data from eight WT vessels showing ~75% reduction in amplitude at most pressures and slight increase in frequency at all pressures except 0.5 cmH_2_O. As in the previous figure, contractions with amplitude < 5 μm were not counted at pressures ≤ 3 cmH_2_O. (**C**) Example recordings from a Ca_v_3.1^−/−^; Ca_v_3.2^−/−^ popliteal lymphatic before and after addition of 100 nM mibefradil to the bath. Mibefradil had a strong inhibitory effect on amplitude. (**D**) Summary data for eight Ca_v_3.1^−/−^; Ca_v_3.2^−/−^ popliteal lymphatics showing pronounced reduction in contraction amplitude at all pressures (no vessels had contractions at 0.5 cmH_2_O and only two vessels had contractions at 1 cmH_2_O (one of those is shown in C). Mibefradil caused an increase in contraction frequency at all pressures above 3 cmH_2_O.

The protocols in Figs. 1 and 5 tested the responses of pressurized lymphatics at a single pressure, compared to the responses at multiple pressures tested (in rat) by Lee *et al*.^35^. Therefore, we performed protocols similar to theirs, in which the contractile parameters of mouse PLs were measured at each pressure between 0.5 and 10 cmH_2_O before and after application of Ni^2+^ (100 μM) or mibefradil (100 nM)—the same concentrations used in their study. The prediction based on their results is that both inhibitors would reduce frequency at most or all pressures but have no effect on amplitude at any pressure. The Ni^2+^ data are shown in Fig. 10 and the mibefradil data are shown in Fig. 11. Contrary to the prediction, our results show that in WT lymphatics both Ni^2+^ and mibefradil inhibited contraction amplitude at all pressures, whereas Ni^2+^ increased frequency at all pressures and mibefradil increased frequency at most pressures (Figs. 10A,B and 11A,B, respectively). The reduction in amplitude with an increase or no change in frequency points to a primary (or exclusive) effect of both inhibitors on L-type channels in mouse lymphatics.

We also repeated these protocols on PLs from Ca_v_3.1^−/−^; Ca_v_3.2^−/−^ double KO mice, reasoning that, if T-type channels were important and the inhibitors were specific for those channels, we would see inhibition of pacemaking frequency in WT vessels but not in Ca_v_3.1^−/−^; Ca_v_3.2^−/−^ vessels. However, as shown in panels C-D of Figs. 10 and 11, the effects of Ni^2+^ and mibefradil were very similar in both WT and Ca_v_3.1^−/−^; Ca_v_3.2^−/−^ PLs; moreover, the most consistent inhibitory effect was on amplitude not frequency. Because the effects of both inhibitors were nearly identical in both genotypes this again suggests primary actions on L-type channels (vessels from both genotypes have L-type channels, but only WT vessels have T-type channels).

### Contractile responses of lymphatics from smooth muscle-specific Ca_v_1.2 KO mice

Next, we hypothesized that deletion of L-type (Ca_v_1.2) channels in the lymphatic smooth muscle layer would unmask a role for Ca_v_3 channels such that Ca_v_3-mediated spontaneous contractions, perhaps weaker ones, would become evident. We reasoned that such events might not be observed in previous studies using nifedipine or other DHPs, even at low concentrations, if those inhibitors had partial inhibitory actions on Ca_v_3 channels. Since global deletion of Ca_v_1.2 is lethal^14^, a conditional tissue-specific knockout strategy was needed. Previously, deletion of Ca_v_1.2 in smooth muscle, using an inducible SM22α-Cre, resulted in mice with lower blood pressure and impaired myogenic constriction in arteries and arterioles^14^. In addition those mice began to die at ~15 days post induction due to a severe loss of GI motility and intestinal impaction^14^. However, those studies did not examine any aspect of lymphatic function. We first checked if the smooth muscle isoform of Ca_v_1.2 was the predominant, or only, isoform expressed by mouse lymphatic vessels. Ca_v_1.2 was expressed in lymphatic smooth muscle based on both PCR (Fig. 2, Fig. S1C) and immunostaining (Suppl. Fig. S11). As shown in Suppl. Fig. S12, Ca_v_1.2a was highly expressed in mouse heart and Ca_v_1.2b was expressed in mouse thoracic aorta and IALs; Ca_v_1.2b was also expressed in brain and heart, presumably due to contamination by blood vessels (panel A). Preliminary analyses failed to reveal possibly unique splice variants of Cav1.2b in lymphatic vessels. The common splice variant exon 9 was found in all samples (panel B), exon 8a and 8b were found in one of the two IAL samples (panel C) and exons 31 and 32 were found in thoracic aorta but in neither of the lymphatic vessel samples (panel D).

**Figure 12:**
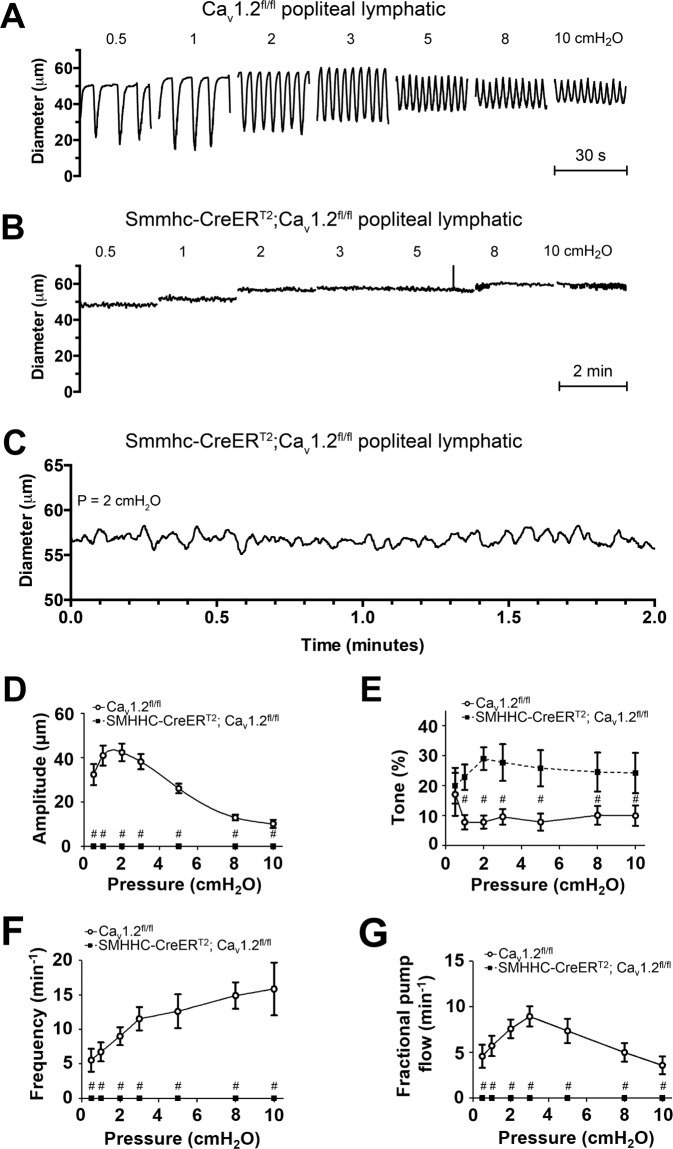
Effects of SM-specific deletion of Ca_v_1.2 on popliteal lymphatic contractile function. (**A**) Contractile patterns in a PL from a Ca_v_1.2 floxed mouse (without Cre) after tamoxifen administration 12 days prior to the experiment. Contraction patterns are essentially identical to those of PLs from WT mice (See Fig. 6A). (**B**) Complete absence of large-amplitude, propulsive contractions in a PL from a SM-specific Ca_v_1.2 KO mouse. The vessel exhibits only very small amplitude vasomotion, which is mostly independent of the pressure level. (**C**) Expanded y-axis scale showing the vasomotion pattern in another PL from a SM-specific Ca_v_1.2 KO mouse. (**D–G**) Summary of contractile parameters for Ca_v_1.2^fl/fl^ (control) and SMMHC-Cre^ERT2^; Ca_v_1.2^fl/fl^ mice studied 10–12 days after tamoxifen administration. With one exception all parameters at all pressures were significantly different between the two groups. The exception was tone at 0.5 cmH_2_O. Note that tone in SM-specific Ca_v_1.2 KO vessels is ~3-fold higher than in the corresponding Ca_v_1.2 floxed controls at every pressure except 0.5 cmH_2_O.

We generated our own smooth muscle-specific, inducible Ca_v_1.2 (Ca_v_1.2b) KO mouse by crossing SMMHCCreER^T2^ mice to Ca_v_1.2(Ca_v_1.2b)^flox^ mice. The tamoxifen induction protocol (daily i.p. injection for 5 days) was optimized first using SMMHCCreER^T2^; Rosa26^mTmG^ reporter mice and revealed >99% recombination in LMCs (not shown). Induction of SMMHCCreER^T2^; Ca_v_1.2^fl/fl^ mice produced animals that began to die between days 12–15 from weight loss and impaction of the intestinal tract. PLs from these mice were therefore studied *ex vivo* 10–12 days after induction. PLs never exhibited any spontaneous contractions larger than 5 μM, even over observation periods of 3–4 hours. Changing pressure over the range 0.5 and 10 cmH_2_O also failed to evoke robust, spontaneous contractions. Example recordings are shown in Fig. 12A,B, where the control vessel (panel A) is from a Ca_v_1.2 ^f/f^ mouse (without Cre) that was injected with tamoxifen for 5 days. The control vessel exhibited robust spontaneous contractions at all pressure levels in contrast to the complete lack of contractions in the SMMHCCreER^T2^; Ca_v_1.2 ^fl/fl^ vessel (panel B). Examination of diameter tracings from the latter vessels at higher resolution showed small, high-frequency fluctuations (panel C) resembling the vasomotion often observed in WT arterioles^80–82^. However, these fluctuations were also observed in PLs from control (both WT and Ca_v_1.2^f/f^) mice and in vessels from Ca_v_3.1^−/−^, Ca_v_3.2^−/−^, and Ca_v_3.1^−/−^; Ca_v_3.2^−/−^ mice (not shown) during the diastolic period of the lymphatic contraction cycle. Fluctuations were more obvious in the smooth muscle-specific Ca_v_1.2 KO mice due to absence of superimposed strong contractions. Calcium imaging of the smooth layer suggested that the small fluctuations corresponded to calcium waves in LMCs (not shown), similar to what has been reported for arteriolar smooth muscle^83,84^. The localized, non-propagating nature of these small diameter fluctuations also distinguished them from the large-amplitude, propagating contractions of WT vessels. Data from 5 PLs from SMMHCCreER^T2^; Ca_v_1.2^fl/fl^ mice are summarized in Fig. 12D–G, where AMP, FREQ and FPF are zero at all pressures, in contrast to the corresponding parameters from 7 PLs from Ca_v_1.2^fl/fl^ mice. Contractions for this (and all other) analyses of FREQ were defined as propagating events larger than 5 μM in amplitude. Interestingly, tone was ~2.5-fold *higher* in these mice, which is opposite to that reported for arteries from SM22aCreER^T2^; Ca_v_1.2^fl/fl^ mice^14^. The Ca_v_1.2 antagonist nifedipine did not alter the level of tone (1 μM, tested at 3 cmH_2_O), nor did the Ca_v_1.2 agonist BayK8644 (1 μM). Neither did Bay K8644 reverse the lack of contractions in smooth muscle-specific Ca_v_1.2 KO mice. In contrast, contractions of (control) Ca_v_1.2^fl/fl^ vessels from mice treated with tamoxifen were slightly enhanced in amplitude by BayK8644 and completely inhibited by nifedipine (Suppl. Fig. S13).

### Characteristics of the resting Vm and APs in LMCs of WT and Ca_v_3.1^−/−^; Ca_v_3.2^−/−^ double KO mice

**Figure 13:**
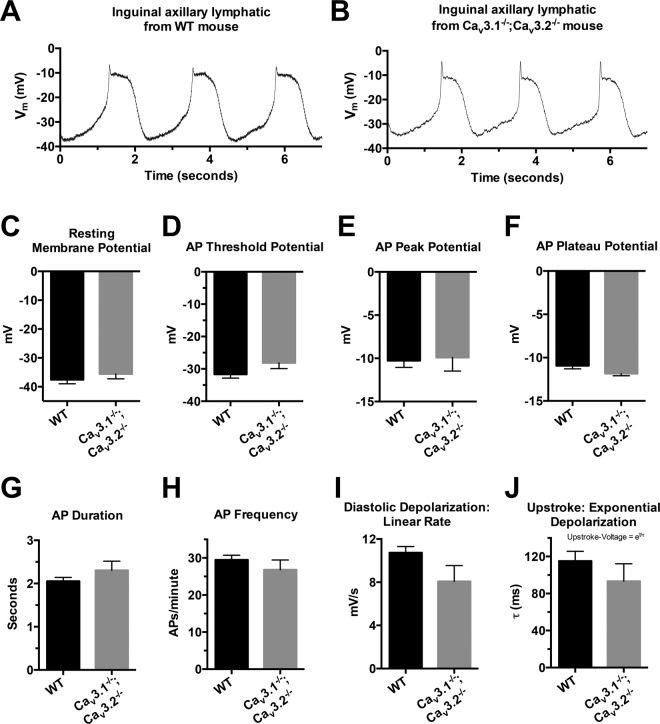
Characteristics of resting Vm and APs in LMCs in the walls of pressurized IALs from WT and Ca_v_3.1^−/−^; Ca_v_3.2^−/−^ double KO mice. (**A,B**) Representative recordings of Vm in the two strains. There was substantial cell to cell variation in the AP spike height, but these recordings are reasonably representative. (**C–J**) Summary of comparisons of resting Vm (**C**) and AP characteristics (**D–J**) as analyzed in the same manner described in ref. ^52^. There were no significant differences in any of the parameters between the two mouse strains, although there were trends for a few parameters (e.g. AP threshold, diastolic depolarization rate, upstroke exponential depolarization time constant) to be lower in Ca_v_3.1^−/−^; Ca_v_3.2^−/−^ vessels.

Finally, we examined whether Ca_v_3 channels might contribute to the AP in lymphatic smooth muscle and be evident in Ca_v_3-deficient vessels as a reduction in the peak or upstroke velocity of the AP. To test this hypothesis sharp electrode recordings of membrane potential (Vm) were made in the LMC layer of pressurized IALs from WT and Ca_v_3.1^−/−^; Ca_v_3.2^−/−^ double KO mice; wortmannin treatment minimized the contraction amplitude so that the electrode would not be dislodged^10^. We previously reported that APs in LMCs from WT mice appear to be initiated around −30 mV by a gradual diastolic depolarization, peak around −5 mV, followed by a plateau lasting 1–2 secs at ~−10 mV^52^. As shown by the example recordings in Fig. 13A,B, the APs from LMCs of WT and Ca_v_3.1^−/−^; Ca_v_3.2^−/−^ vessels were remarkably similar in appearance. Because the peak of the AP and the upstroke velocity are sensitive to even partial inhibition of whatever voltage-gated cation channel carries current (Na_v_, Ca_v_3, Ca_v_1, etc.), we compared those parameters, and others, of the AP in lymphatic smooth muscle from WT and Ca_v_3.1^−/−^; Ca_v_3.2^−/−^ vessels (Fig. 13C–J). No parameters of the AP or resting Vm were significantly different between WT and Ca_v_3.1^−/−^; Ca_v_3.2^−/−^ vessels. These results suggest that Ca_v_3 channels do not significantly affect the upstroke velocity or the peak of the AP (i.e. characteristics of the spike), which is what might have been expected. Diastolic depolarization rate tended to be lower in Ca_v_3.1^−/−^; Ca_v_3.2^−/−^ than WT vessels, but that change did not result in a significant change in the pacemaking frequency.

## Discussion

The aim of this study was to test the hypothesis that T-type VGCCs play an important role in modulating the frequency of lymphatic vessel pacemaking. We confirmed the expression of two Ca_v_3 channel isoforms (Ca_v_3.1, Ca_v_3.2) by RT-PCR in rat mesenteric lymphatic smooth muscle. We confirmed previous findings that the FREQ, but not AMP, of spontaneous contractions of rat mesenteric lymphatics was sensitive to inhibition by mibefradil, although we were unable to confirm a similar, selective inhibitory effect of Ni^2+^ on the FREQ of rat mesenteric lymphatics. In the mouse, we demonstrated the expression of Ca_v_3.1 and Ca_v_3.2 in popliteal and IAL lymphatic smooth muscle by RT-PCR and immunofluorescence and verified the presence of functional VGCCs by patch clamp protocols, in which the kinetics and nifedipine resistance of whole-cell Ca^2+^ currents were consistent with those of Ca_v_3 channels. In both popliteal and IALs of the mouse, Ni^2+^ had equivalent inhibitory effects on both the FREQ and AMP of spontaneous contractions. The equivalent IC_50_ values for Ni^2+^, coupled with the likelihood that L-type channels were also inhibited at those concentrations, led us to explore genetic deletion methods to more selectively test the role of Ca_v_3 channels in lymphatic smooth muscle. We tested two previously-documented global knock-out models, Ca_v_3.1^−/−^ and Ca_v_3.2^−/−^ mice, and also generated the first double KO Ca_v_3.1^−/−^; Ca_v_3.2^−/−^ mouse, assessing the contractile properties of both popliteal and IAL vessels in each mouse model. We also generated and tested a smooth-muscle specific KO of the L-type calcium channel, Ca_v_1.2. Spontaneous contractions were completely absent from lymphatic vessels from Ca_v_1.2-deficient mice, and they could not be evoked by pressure changes. This finding indicates that Ca_v_1.2 channels are obligatory for spontaneous twitch contractions. In contrast, none of the contractile parameters tested, including FREQ and AMP, were significantly different in any of the three Ca_v_3 KO mouse strains compared to those of WT vessels. This finding held up for both popliteal and IAL vessels at all pressures tested (from 0.5 to 10 cmH_2_O), with the only exceptions being an *accentuated* AMP at a few selective pressures in some Ca_v_3-deficient vessels. Comparisons of the effects of channel deletion with the effects of Ni^2+^ and mibefradil suggest that previous conclusions concerning a role for Ca_v_3 channels in LMCs using so-called selective T-type channel inhibitors likely are explained by off-target effects on L-type calcium channels. This conclusion is further strengthened by the results of protocols in which we compared the effects of reputedly selective concentrations of mibefradil and Ni^2+^ on WT and Ca_v_3.1^−/−^; Ca_v_3.2^−/^ lymphatic vessels. Ideally, if mibefradil and Ni^2+^ selectively inhibited T-type channels, neither compound would have an effect on Ca_v_3.1^−/−^; Ca_v_3.2^−/−^ vessels (which lack T-type channels), but they would decrease pacemaking frequency in WT vessels without any change in amplitude. However in both genotypes both compounds produced a profound impairment of contraction amplitude and a slight increase in pacemaking frequency at almost all pressures. This result further reinforces our conclusion that these compounds primarily or exclusively inhibit L-type VGCCs in mouse lymphatic vessels. Finally, we measured membrane potentials and action potentials in the smooth muscle layer of mouse IAL vessels and found slight, but not significant, differences in the resting Vm, the duration of the AP plateau and the rate of diastolic depolarization and upstroke velocity in Ca_v_3 DKO vessels compared to WT vessels. We conclude that these differences in the characteristics of the AP are too subtle to result in significant differences in the amplitude or frequency of spontaneous lymphatic contractions.

Our work is the first report of a lymphatic phenotype in smooth-muscle-specific Ca_v_1.2 KO mice. We engineered this mouse to avoid possible and subtle off-target effects of nifedipine on Ca_v_3 channels, which are known to occur with recombinant Ca_v_3 channels^85^. Lymphatic vessels from smooth muscle-specific Ca_v_1.2 KO mice have no spontaneous, propagated contractions whatsoever and contractions could not be stimulated by multiple interventions, including input and/or output pressure elevation. Thus, in the absence of Ca_v_1.2 channels, Ca_v_3 channels are insufficient to evoke APs and propulsive contractions in mouse LMCs, which is further confirmation of a dominant role for Ca_v_1.2 channels. Interestingly, lymphatic tone in smooth muscle-specific Ca_v_1.2 KO vessels was elevated 3-fold over that of control vessels, which is opposite to that observed in arteries of SM22aCreER^T2^; Ca_v_1.2 ^f/f^ mice^14^; the subsequent elimination of that tone in Ca^2+^ free Krebs solution implies that lymphatic smooth muscle tone is strongly regulated by VGCC-independent calcium-influx mechanisms, perhaps through Rho kinase^86^, and perhaps even being *attenuated* by VGCC-dependent Ca^2+^ influx.

If functional Ca_v_3 channels are expressed in mouse LMCs, why does their absence neither affect the AP in LMCs in an obvious way nor alter the characteristics of the spontaneous twitch contraction? Based on numerous studies of native and recombinant Ca_v_3 channels, the typical activation threshold for Ca_v_3 channels is −70 mV^17^, with a current window between −65 and −45 mV^87,88^; in comparison, the current window for L-type VGCCs is typically between −30 and 0 mV^89^. At the resting Vm that we measured in mouse LMCs (~−35 mV) (Fig. 13 and ref. ^52^), it is likely that Ca_v_3 channels are completely inactivated. Because Vm values in rat and human mesenteric LMCs are only slightly more negative (−40 and −45 mV, respectively; ref. ^90^ and unpublished observations in pressurized human mesenteric lymphatics), a similar degree of inactivation is likely to prevail in lymphatic vessels of those species (unless a more right-shifted splice variant of Ca_v_3.1 is expressed^91^). It is possible, however, if LMCs were chronically hyperpolarized, e.g. by a vasoactive agent, that rapid depolarization to threshold would recruit Ca_v_3 channels to participate in a subsequent AP. This hypothesis remains to be tested.

In mouse LMCs, Ca_v_3 channels do not appear to play any detectable role in mediating the inhibition of spontaneous lymphatic contractions by ACh: we could find no significant differences in the concentration-dependent inhibition by ACh in Ca_v_3-deficient vessels compared to WT vessels. A possible role for Ca_v_3 channels in arterial endothelium was suggested by Figueroa *et al*.^67^ who hypothesized that an electrically-induced signal could be conducted along the arterial endothelium and sequentially activate both Ca_v_3.2 and Na_v_ channels to produce Ca^2+^ influx that would subsequently regulate NO production. Gilbert *et al*.^92^ found evidence that Ca_v_3.1 channels in mouse pulmonary endothelium contribute to ACh-induced [Ca^2+^] increases that drive NO production. This idea, however, is highly controversial^93,94^ in large part due to lack of solid electrophysiological evidence for the expression of functional Ca_v_3 or Na_v_ (or Ca_v_1) channels in intact endothelium or native endothelial cells. In confirmation of the skeptical view we were unable to detect either Ca_v_3.1 or Ca_v_3.2 (or Ca_v_1.2) message in pure LEC tubes. Neither could we find a consistent impairment in ACh-induced inhibition between Ca_v_3.1^−/−^, Ca_v_3.2^−/−^ or Ca_v_3.1^−/−^; Ca_v_3.2^−/−^ vessels and WT vessels **(**Suppl. Figs. S7–S10). Previously we showed that ACh-induced inhibition of mouse popliteal lymphatic contractions was solely dependent on NO^55^. If Ca_v_3 channels were an important target of LEC-derived NO, as shown for some blood vessels^21,75^, we would have expected the ACh concentration-response relationships for AMP or FREQ, or both, to be right-shifted compared to WT vessels. Although there was a trend for this to occur in Ca_v_3.1^−/−^ vessels, the differences were not significant. In cerebral artery smooth muscle cells Ca_v_3.2 channels colocalize with RYR2 to microdomains and their blockade also disrupts RYR-mediated spark production and subsequent BK channel activation that normally counters cerebral arterial constriction^23,33^. Our data do not directly address this issue; however, a role for Ca^2+^ sparks in lymphatic smooth muscle, or even their occurrence, has not been demonstrated^7^. Moreover, ryanodine is reportedly without any effect on guinea pig lymphatic smooth muscle^10^ although its actions in mouse lymphatic smooth muscle are currently being investigated with genetic tools.

Despite the analysis above, it would be premature to conclude that Ca_v_3 channels have no important role whatsoever in lymphatic function. The observations that two different Ca_v_3 isoforms are expressed and can be activated by depolarization (under voltage clamp conditions) suggest that they *should likely* participate in some aspect of lymphatic function. We can conclude from our data only that Ca_v_3 channels play no overt role in normal regulation of the amplitude or frequency of spontaneous twitch contractions in lymphatic vessels of the mouse nor are they required for ACh-mediated inhibition of those contractions. However, we cannot rule out the possibility that Ca_v_3 channels might play a more substantial role in spontaneous contractions of lymphatic vessels from other species, or that they may be critical for other aspects of lymphatic function even in the mouse, including responses to agonists other than ACh, to nerve stimulation, for remodeling, or mediating immune cell interactions. These possibilities, and others, await to be explored in future studies.

The question of whether T-type calcium channels play a role in lymphatic function is relevant to human medicine because of the possibility for therapeutic targeting of dysfunctional lymphatic vessels in chronic lymphedema. Olszewski’s observations of patients with impaired lymphatic smooth muscle contraction strength and/or pacemaking in chronic lymphedema^46–48^ point to a problem involving an ionic mechanism that potentially might be corrected pharmacologically. However, eventual therapeutic targeting of ionic dysfunction in human LMCs will require knowledge of the specific VGCC isoforms expressed in humans as well as the development of selective inhibitors to block those channels. Whether Ca_v_3 channels are expressed and are critical to some aspect of lymphatic function in humans remains unknown.

## Supplementary information

Supplemental Figures.

## Data Availability

The datasets analyzed during the current study are available from the corresponding author on reasonable request.
