# Supplementary material for: T-type, but not L-type, voltage-gated calcium channels are dispensable for lymphatic pacemaking and spontaneous contractions
Source: Sci Rep. 2020 Jan 9;10:70. doi: 10.1038/s41598-019-56953-3 (PMC6952455; doi:10.1038/s41598-019-56953-3)
Supplement: Supplementary file 1 — Supplemental Figures. [file 41598_2019_56953_MOESM1_ESM.pdf]

# Supplementary Information

## T-type, but not L-type, voltage-gated calcium channels are dispensable for lymphatic pacemaking and spontaneous contractions

Kim H. T. To, Peichun Gui, Min Li, Scott D. Zawieja, Jorge A. Castorena-Gonzalez, and Michael J. Davis

Department of Medical Pharmacology and Physiology, University of Missouri School of Medicine, Columbia, Missouri 65212

Contents:

1. Supplemental Figures S1-S13.

2. Supplemental Figure legends.

**Figure S1**

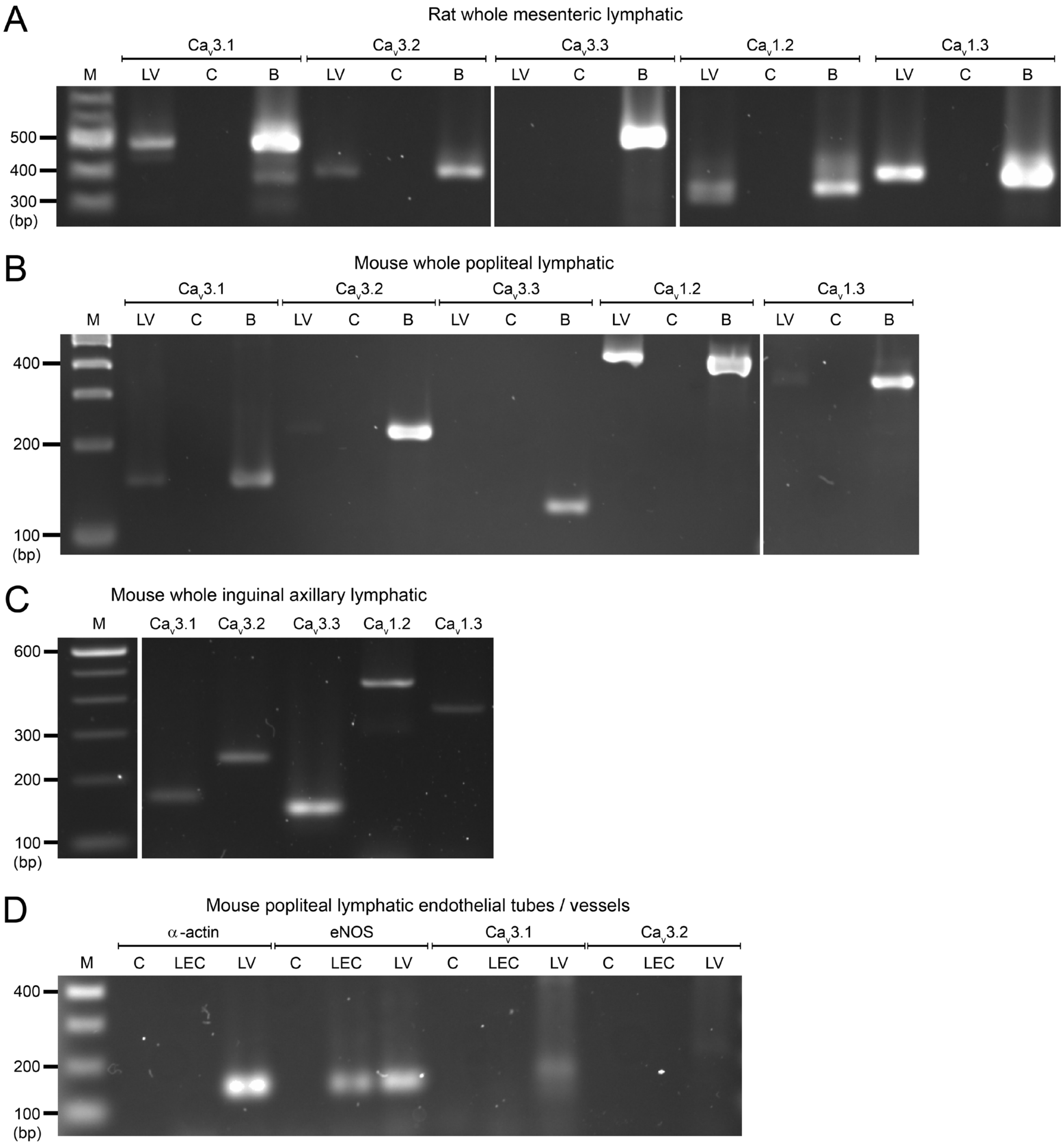
A
Rat whole mesenteric lymphatic
Cav3.1
Cav3.2
Cav3.3
Cav1.2
Cav1.3
M
LV C B LV C B LV C B LV C B LV C B
500
400
300
(bp)
B
Mouse whole popliteal lymphatic
Cav3.1
Cav3.2
Cav3.3
Cav1.2
Cav1.3
M
LV C B LV C B LV C B LV C B LV C B
400
200
100
(bp)
C
Mouse whole inguinal axillary lymphatic
M
Cav3.1
Cav3.2
Cav3.3
Cav1.2
Cav1.3
600
300
200
100
(bp)
D
Mouse popliteal lymphatic endothelial tubes / vessels
α-actin
eNOS
Cav3.1
Cav3.2
M
C LEC LV C LEC LV C LEC LV C LEC LV
400
200
100
(bp)

Figure S2

**A** **WT IAL pretreated with antigenizing peptide for $Ca_v3.1$ antibody**

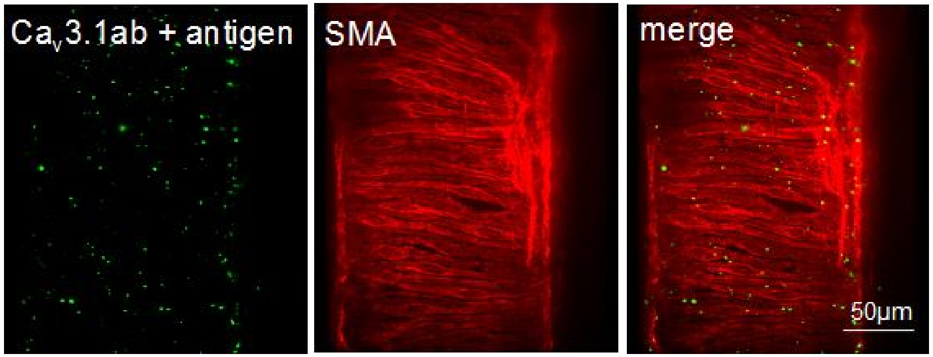


**B** **Cav3.1$^{-/-}$ IAL**

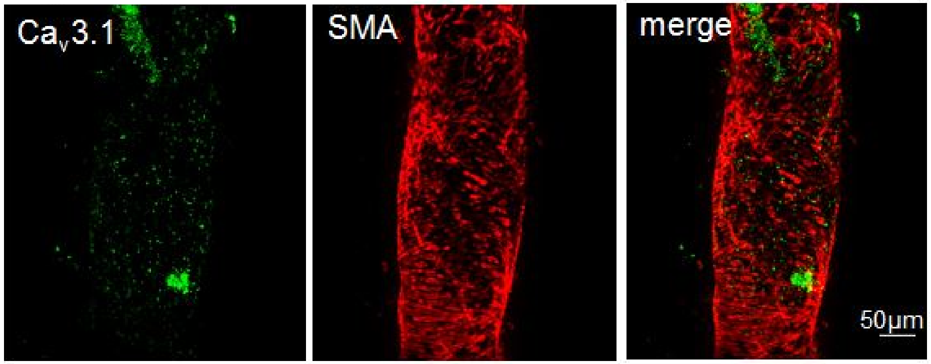


**C** **WT mesenteric artery**

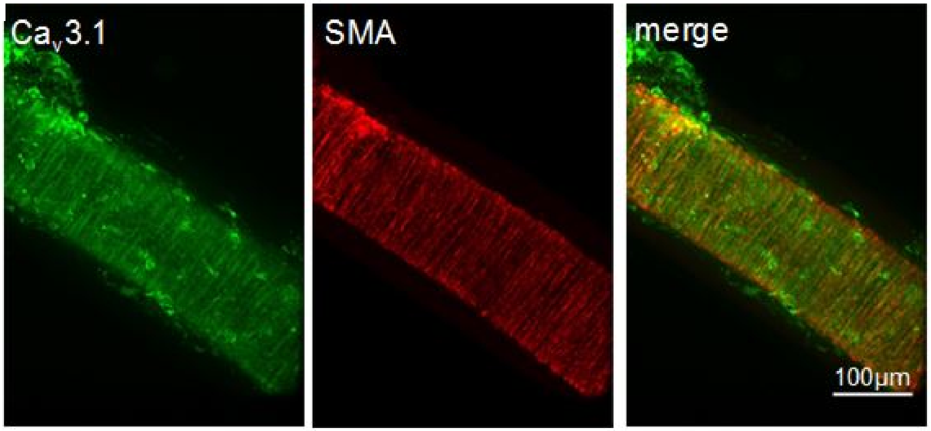

**Figure S3**

**A WT IAL pretreated with antigenizing peptide for $Ca_v3.2$ antibody**

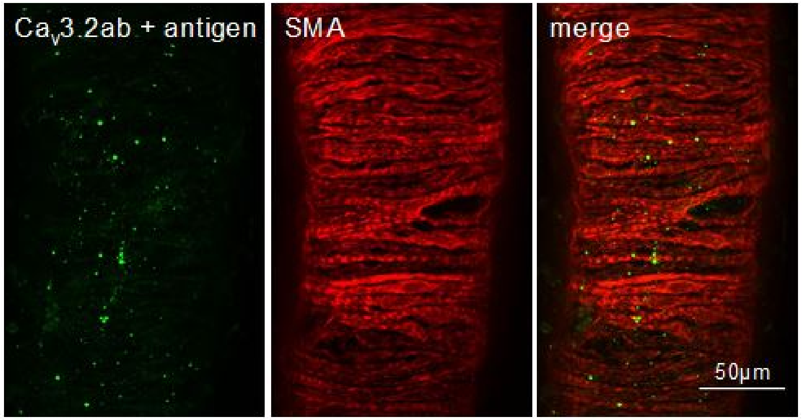


**B $Cav3.2^{-/-}$ IAL**

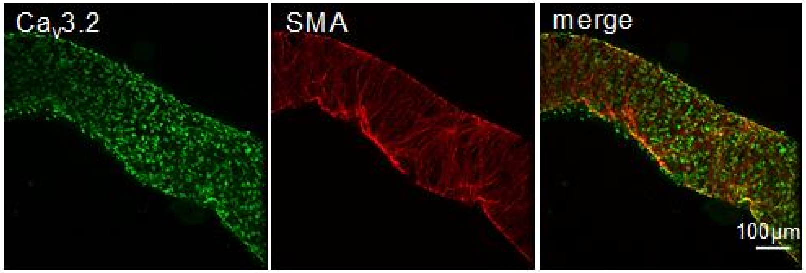


**C WT mesenteric artery**

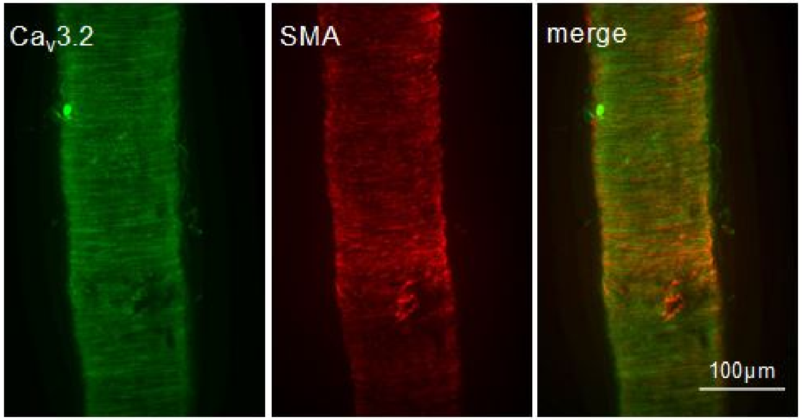

**Figure S4**

**A** **$Ni^{2+}$-sensitive VGCC current in mouse popliteal LSMC**

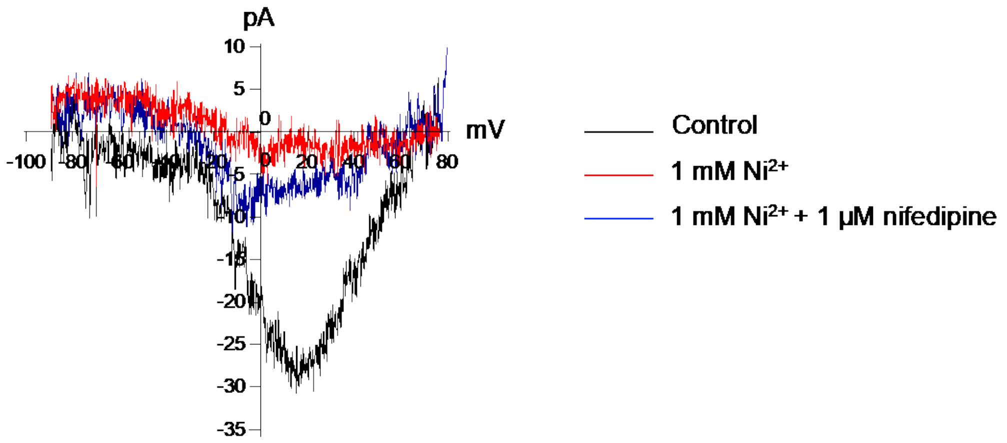


**B** **$Ni^{2+}$-sensitive VGCC current in rat mesenteric LSMC**

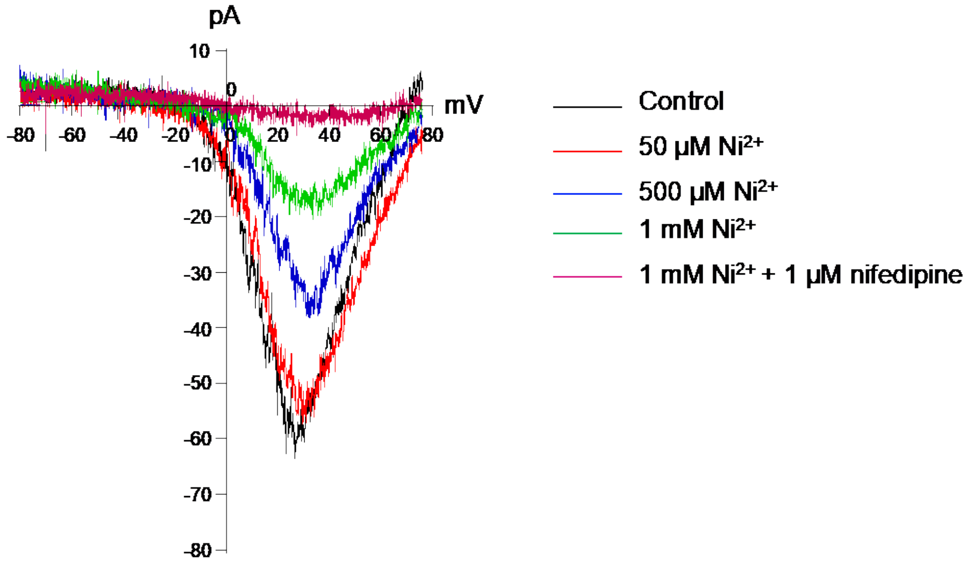

Figure S5

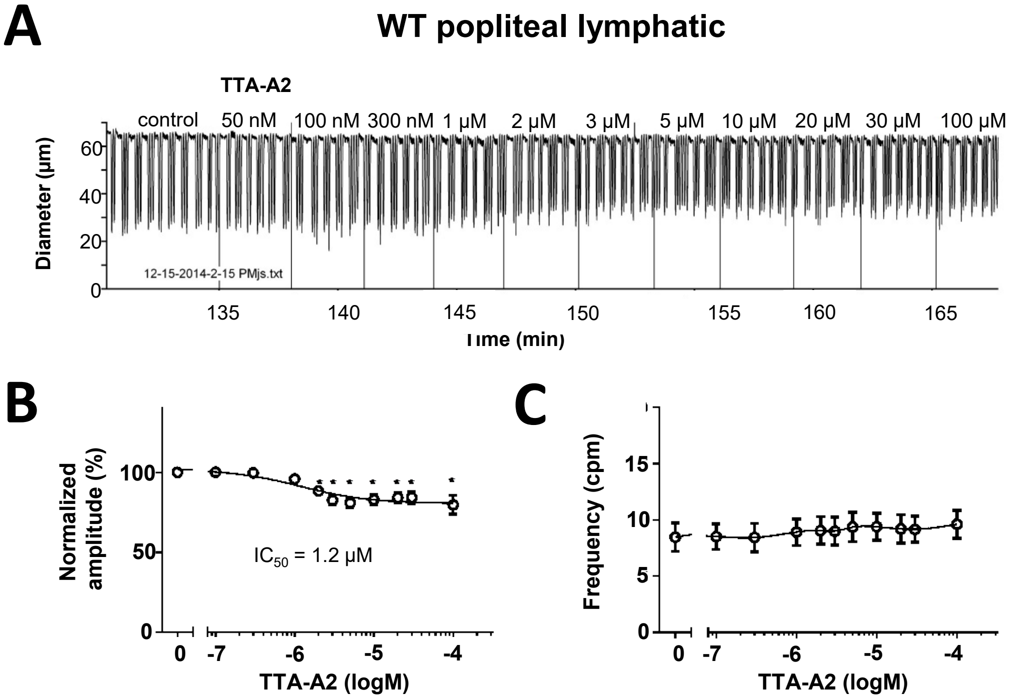
A
WT popliteal lymphatic
TTA-A2
control
50 nM
100 nM
300 nM
1 μM
2 μM
3 μM
5 μM
10 μM
20 μM
30 μM
100 μM
Diameter (μm)
12-15-2014-2-15 PMjs.txt
Time (min)
B
Normalized amplitude (%)
IC50 = 1.2 μM
TTA-A2 (logM)
C
Frequency (cpm)
TTA-A2 (logM)

**Figure S6**

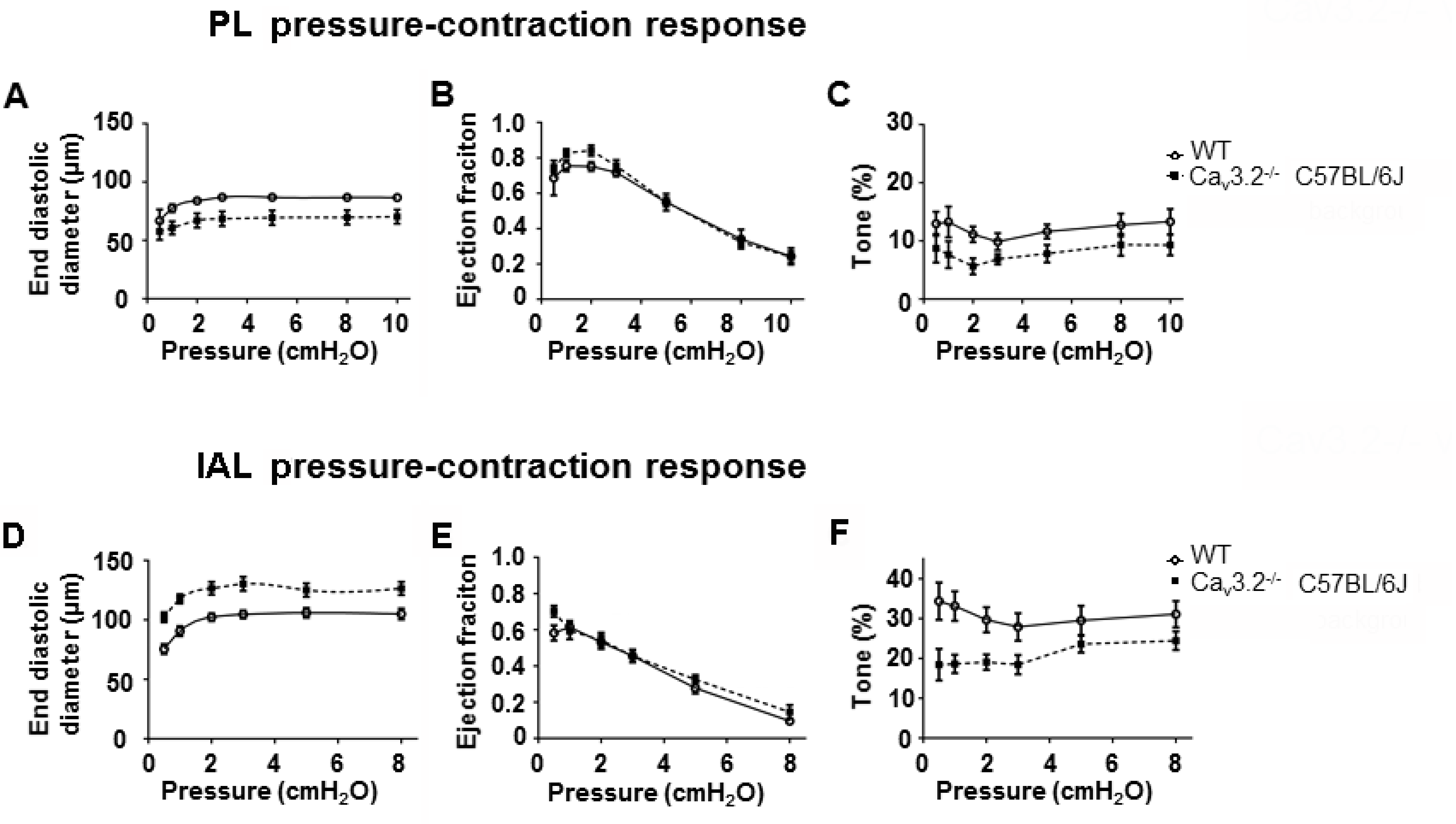
PL pressure-contraction response
A
End diastolic diameter (µm)
150
100
50
0
0 2 4 6 8 10
Pressure (cmH2O)
B
Ejection fraciton
1.0
0.8
0.6
0.4
0.2
0
0 2 4 6 8 10
Pressure (cmH2O)
C
Tone (%)
30
20
10
0
0 2 4 6 8 10
Pressure (cmH2O)
WT
Cav3.2-/- C57BL/6J
IAL pressure-contraction response
D
End diastolic diameter (µm)
150
100
50
0
0 2 4 6 8
Pressure (cmH2O)
E
Ejection fraciton
1.0
0.8
0.6
0.4
0.2
0
0 2 4 6 8
Pressure (cmH2O)
F
Tone (%)
40
30
20
10
0
0 2 4 6 8
Pressure (cmH2O)
WT
Cav3.2-/- C57BL/6J

**Figure S7**

## Pressure-contraction response (PL Cav3.2$^{-/-}$ mixed)

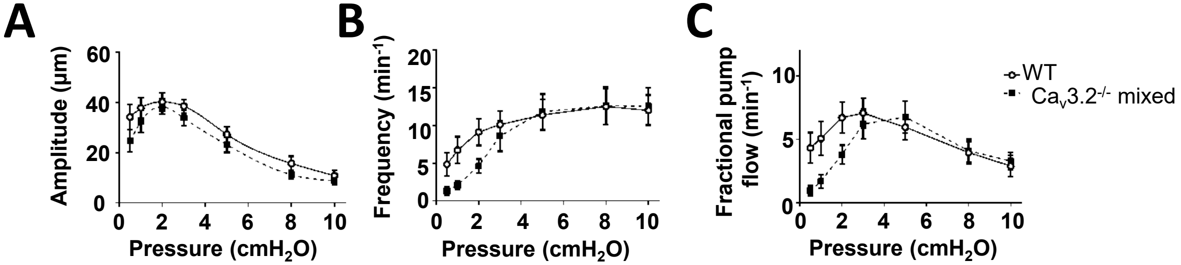


## Acetylcholine dose-response inhibition (PL Cav3.2$^{-/-}$ mixed)

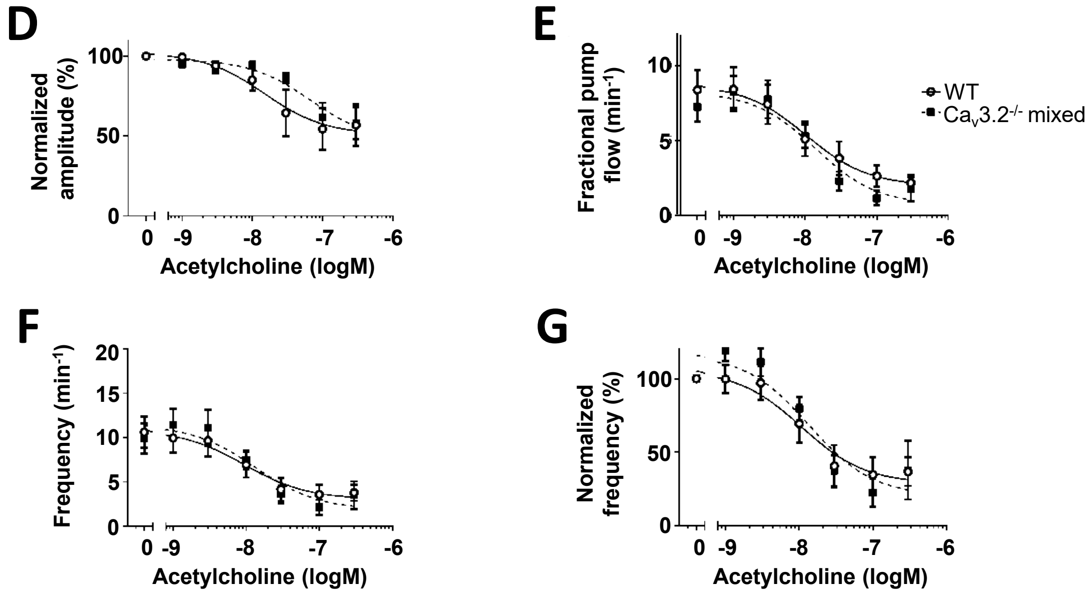

Figure S8

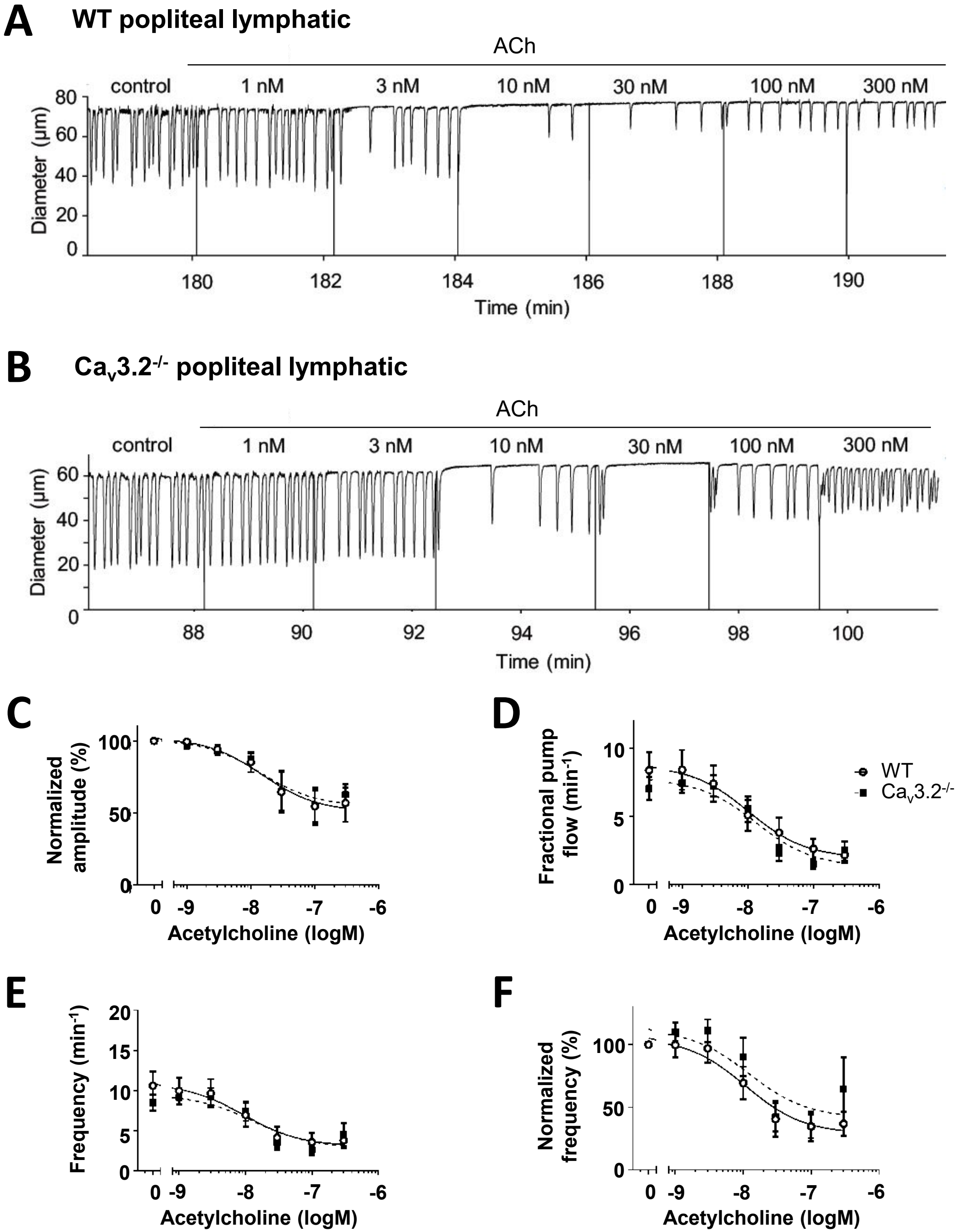

A
WT popliteal lymphatic
ACh
control
1 nM
3 nM
10 nM
30 nM
100 nM
300 nM
Diameter (µm)
Time (min)
B
Ca$_v$3.2$^{-/-}$ popliteal lymphatic
ACh
control
1 nM
3 nM
10 nM
30 nM
100 nM
300 nM
Diameter (µm)
Time (min)
C
Normalized amplitude (%)
Acetylcholine (logM)
D
Fractional pump flow (min$^{-1}$)
Acetylcholine (logM)
WT
Ca$_v$3.2$^{-/-}$
E
Frequency (min$^{-1}$)
Acetylcholine (logM)
F
Normalized frequency (%)
Acetylcholine (logM)

**Figure S9**

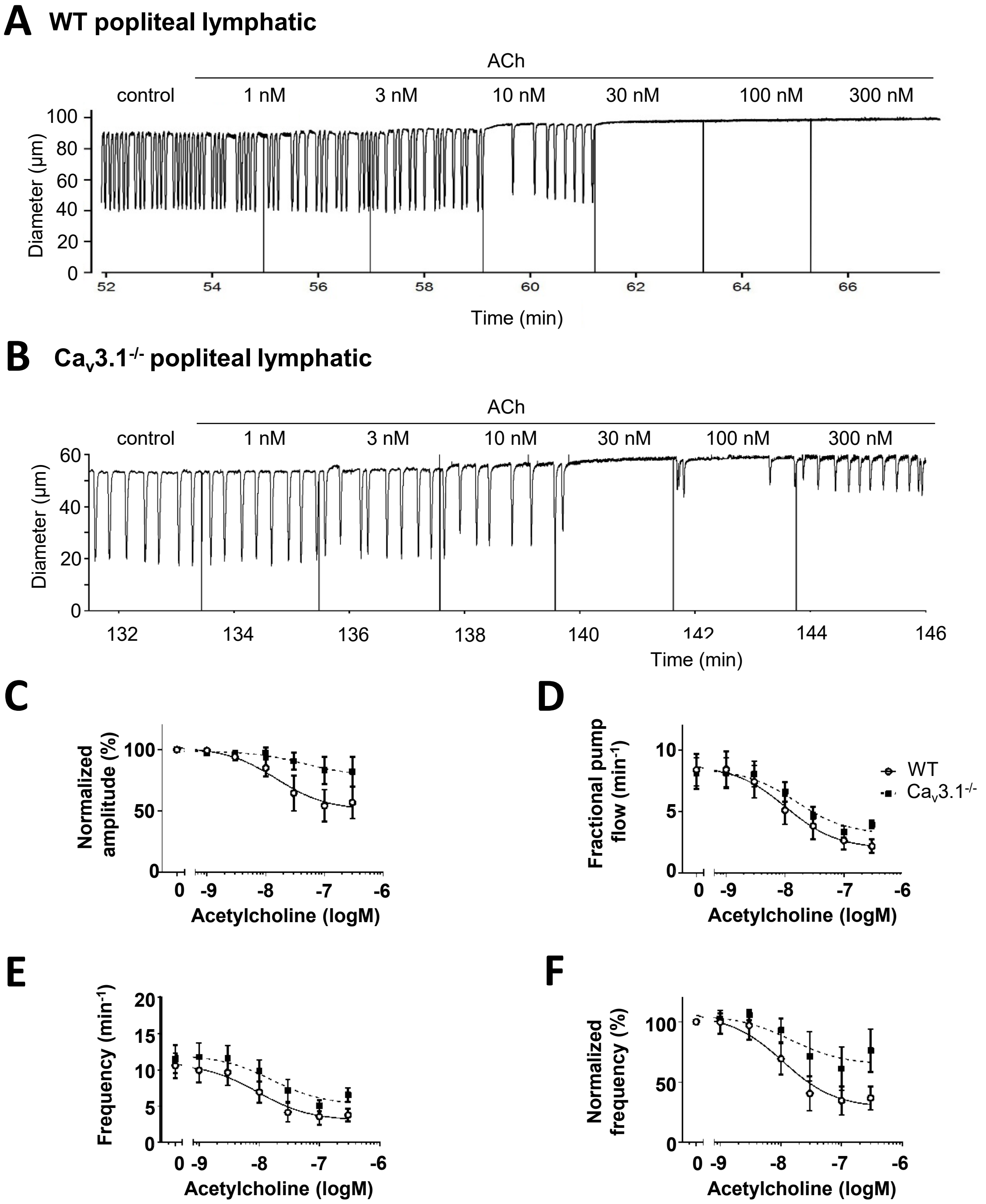
A
WT popliteal lymphatic
ACh
control
1 nM
3 nM
10 nM
30 nM
100 nM
300 nM
Diameter (µm)
Time (min)
B
Ca$_v$3.1$^{-/-}$ popliteal lymphatic
ACh
control
1 nM
3 nM
10 nM
30 nM
100 nM
300 nM
Diameter (µm)
Time (min)
C
Normalized amplitude (%)
Acetylcholine (logM)
D
Fractional pump flow (min$^{-1}$)
Acetylcholine (logM)
WT
Ca$_v$3.1$^{-/-}$
E
Frequency (min$^{-1}$)
Acetylcholine (logM)
F
Normalized frequency (%)
Acetylcholine (logM)

**Figure S10**

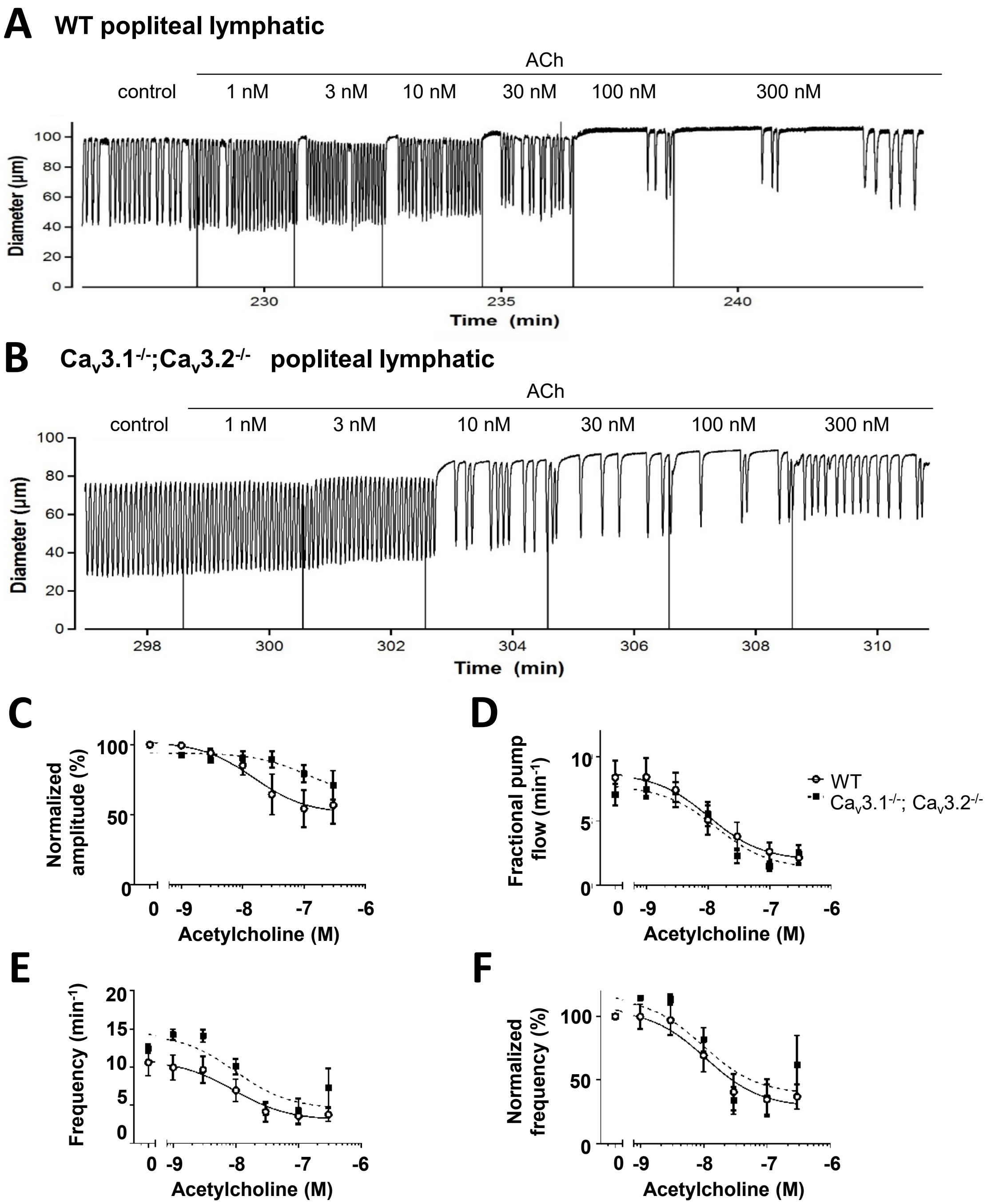
A
WT popliteal lymphatic
ACh
control
1 nM
3 nM
10 nM
30 nM
100 nM
300 nM
Diameter (µm)
Time (min)
B
$Ca_v3.1^{-/-};Ca_v3.2^{-/-}$ popliteal lymphatic
ACh
control
1 nM
3 nM
10 nM
30 nM
100 nM
300 nM
Diameter (µm)
Time (min)
C
Normalized amplitude (%)
Acetylcholine (M)
D
Fractional pump flow ($min^{-1}$)
Acetylcholine (M)
WT
$Ca_v3.1^{-/-}$; $Ca_v3.2^{-/-}$
E
Frequency ($min^{-1}$)
Acetylcholine (M)
F
Normalized frequency (%)
Acetylcholine (M)

Figure S11

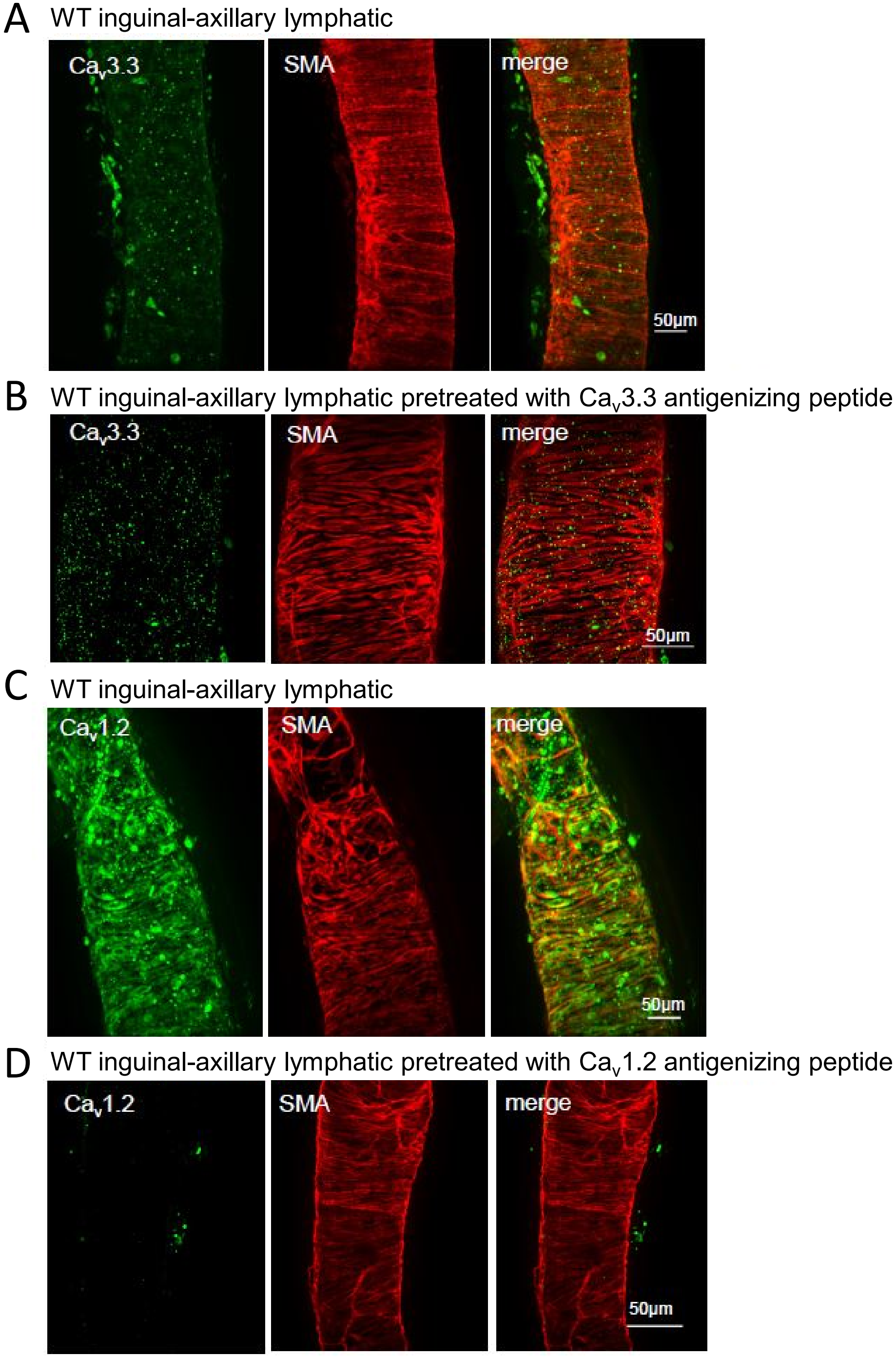
A
WT inguinal-axillary lymphatic
$Ca_v3.3$
SMA
merge
50µm
B
WT inguinal-axillary lymphatic pretreated with $Ca_v3.3$ antigenizing peptide
$Ca_v3.3$
SMA
merge
50µm
C
WT inguinal-axillary lymphatic
$Ca_v1.2$
SMA
merge
50µm
D
WT inguinal-axillary lymphatic pretreated with $Ca_v1.2$ antigenizing peptide
$Ca_v1.2$
SMA
merge
50µm

**Figure S12**

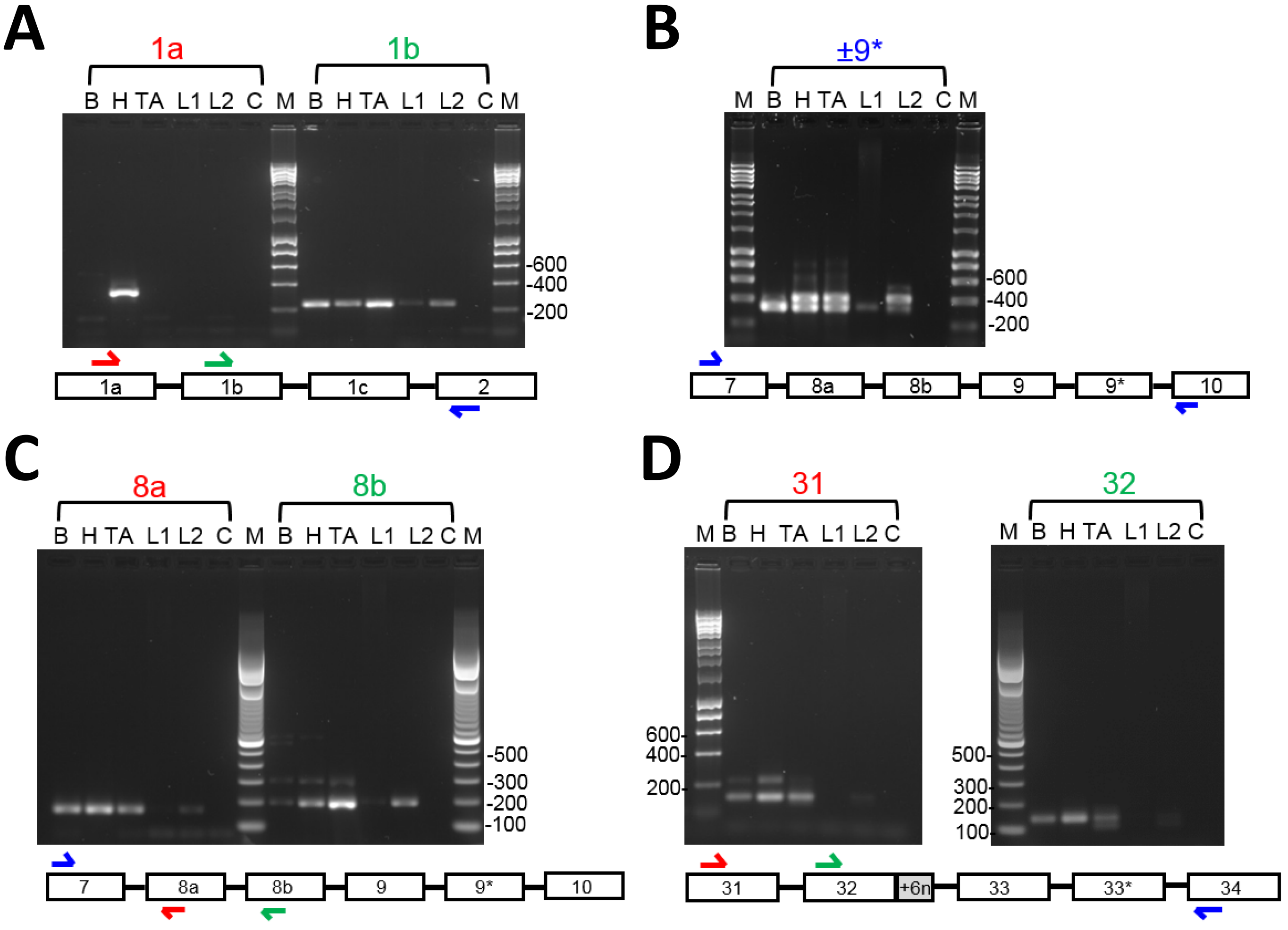


Predicted PCR products

| | |
|---|---|
| Exon 1a | 324 bp |
| Exon 1b | 234 bp |
| ±Exon 9* | 339 bp±414 bp |

B: brain
H: heart
TA: thoracic aorta
L1: lymphatic
L2: lymphatic
C: control ($H_2O$)

Predicted PCR products

| | |
|---|---|
| Exon 8a | 155 bp |
| Exon 8b | 190 bp |
| Exon 31 | 136 bp |
| Exon 32 | 130 bp |

**Figure S13**

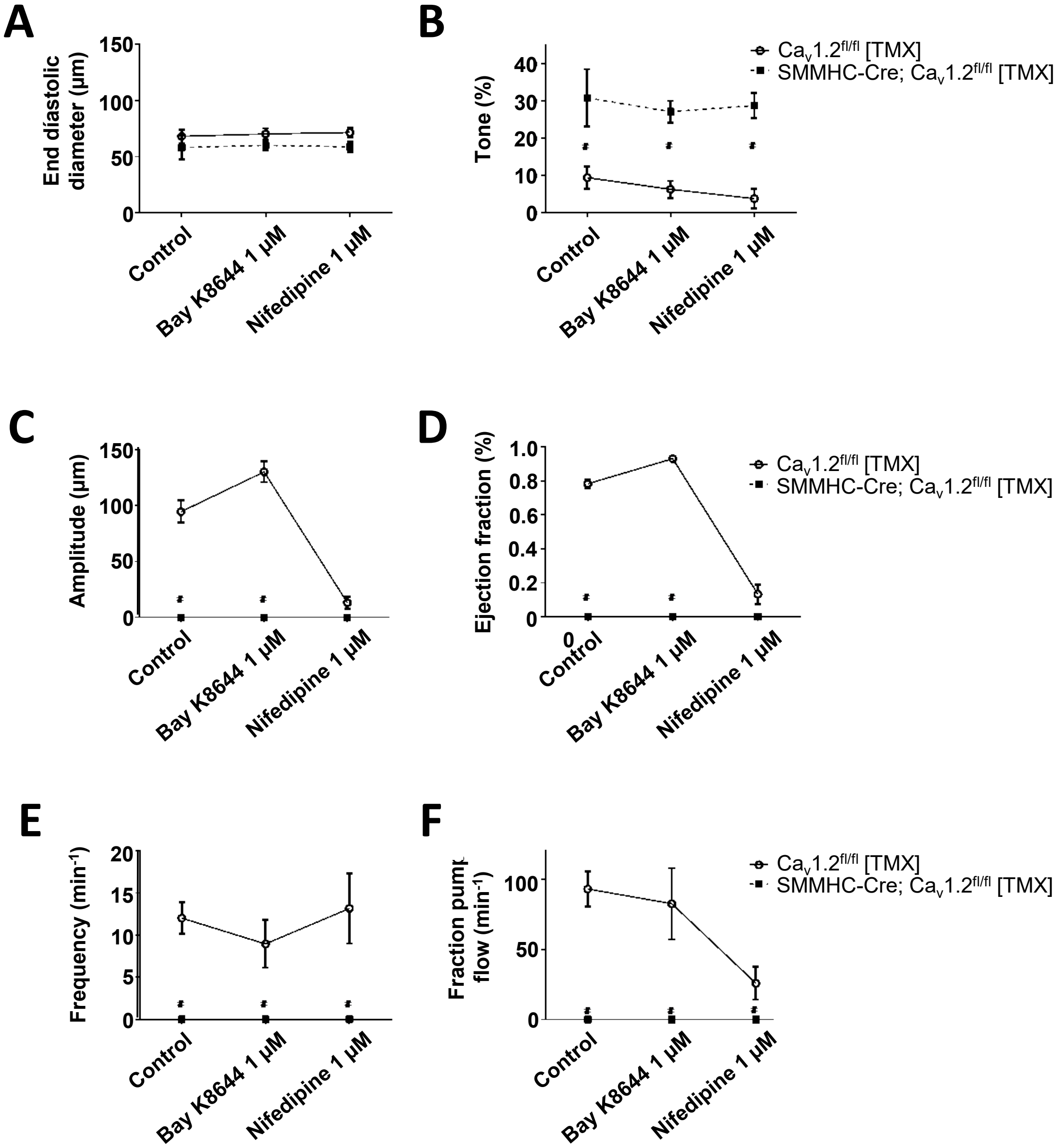

A
End diastolic diameter (µm)
150
100
50
0
Control
Bay K8644 1 µM
Nifedipine 1 µM
B
Tone (%)
40
30
20
10
0
Control
Bay K8644 1 µM
Nifedipine 1 µM
Cav1.2fl/fl [TMX]
SMMHC-Cre; Cav1.2fl/fl [TMX]
C
Amplitude (µm)
150
100
50
0
Control
Bay K8644 1 µM
Nifedipine 1 µM
D
Ejection fraction (%)
1.0
0.8
0.6
0.4
0.2
0
Control
Bay K8644 1 µM
Nifedipine 1 µM
Cav1.2fl/fl [TMX]
SMMHC-Cre; Cav1.2fl/fl [TMX]
E
Frequency (min-1)
20
15
10
5
0
Control
Bay K8644 1 µM
Nifedipine 1 µM
F
Fraction pump flow (min-1)
100
50
0
Control
Bay K8644 1 µM
Nifedipine 1 µM
Cav1.2fl/fl [TMX]
SMMHC-Cre; Cav1.2fl/fl [TMX]
